# HMGB1 expression in CT26 murine cancer cells modulates the tumor microenvironment and limits local anti-tumor immunity

**DOI:** 10.1007/s00018-026-06334-7

**Published:** 2026-08-04

**Authors:** Betül Taşkoparan, Viola Puddinu, Bart Boersma, Lorenzo Spagnuolo, Hélène Poinot, Eloise Dupuychaffray, Montserrat Alvarez, Muriel Urwyler, Marietta Margareta Korsos, Darko Stojkov, Aurélien Pommier, Paul R. Walker, Carole Bourquin

**Affiliations:** 1 School of Pharmaceutical Sciences, University of Geneva, Geneva, Switzerland; 2 Institute of Pharmaceutical Sciences of Western Switzerland, University of Geneva, Geneva, Switzerland; 3 Institute of Pharmacology, University of Bern, Bern, Switzerland; 4 Translational Research Centre in Oncohaematology, Faculty of Medicine, University of Geneva, Geneva, Switzerland; 5 Department of Anaesthetics, Pharmacology, Intensive Care and Emergencies, Faculty of Medicine, University of Geneva, Geneva, Switzerland

**Keywords:** High-mobility group box 1, Damage associated molecular pattern, Cancer, Cancer immunity, Tumor microenvironment

## Abstract

High mobility group box protein 1 (HMGB1) is a ubiquitous protein that has multiple functions in the nucleus and cytosol. Following release into the extracellular space, HMGB1 acts as a mediator of inflammation. HMGB1 is overexpressed in many cancers and has both oncogenic and antitumoral effects. Here we investigated the role of HMGB1 expression by cancer cells in the antitumor immune response. Subcutaneous tumors were induced in mice by injection of wild-type or HMGB1-low engineered syngeneic cancer cells. Tumor growth and immune infiltration were assessed, and gene expression was analysed in whole tumors and isolated tumor cells by RNA sequencing analysis. Downregulation of cancer cell-intrinsic HMGB1 prevented tumor growth and reprogrammed the tumor microenvironment into a more inflamed phenotype. Tumor growth was not impacted in immunodeficient mice or in mice deficient in CD8^+^ T cells, demonstrating that the impact of HMGB1 downregulation on tumor progression was immune-dependent and mediated by CD8^+^ T cells. Transcriptional profiling showed that HMGB1^low^ tumors were enriched in immune pathways such as antigen presentation, T-cell receptor and cytokine signalling. Immune activation was robust and detectable in both tumor tissue and secondary lymphoid organs of mice bearing HMGB1^low^ tumors, but this did not facilitate immune infiltration of wild-type tumors that had been co-injected in the contralateral flank. We propose that tumor cell-intrinsic HMGB1 plays a critical role in modulating the local tumor microenvironment and in limiting anticancer immune responses. Thus, targeting cancer cell-intrinsic HMGB1 may represent a novel approach to improve the outcome of cancer immunotherapy.

**Supplementary Information:**

The online version contains supplementary material available at https://doi.org/10.1007/s00018-026-06334-7.

## Introduction

In cancer, a key determinant of the clinical outcome is the degree of immune cell infiltration, particularly the abundance of cytotoxic T cells [1, 2]. In highly infiltrated tumors, antigen presentation and T-cell activation are enhanced, and secretion of chemokines such as CXCL9 and CXCL10 allows activated cytotoxic T cells to infiltrate the tumor site. Such a pre-existing immune response within the tumor is essential for effective immunotherapy [3]. Understanding how cancer cells shape their microenvironment and in particular how tumor-intrinsic factors regulate the degree of immune cell infiltration is an essential step towards the further development of effective immunotherapeutic strategies for cancer patients.

Here we have studied the impact of cancer cell-intrinsic high mobility group box 1 (HMGB1) protein on tumor progression and on development of an antitumor immune response. HMGB1 is a highly conserved, ubiquitously expressed protein with multiple intracellular and extracellular functions, including transcriptional regulation, promotion of autophagy and inflammation, and cell migration [4]. Under homeostatic conditions, HMGB1 is expressed in the nucleus and functions as a structural protein for chromatin [5]. In contrast, under stress conditions such as injury, inflammation, or infection, HMGB1 is either passively released by dying cells, including cancer cells, or actively secreted by immune cells upon a variety of stimuli. The released HMGB1 then acts as a danger-associated molecular pattern (DAMP), binding to a panel of receptors and activating pro-inflammatory pathways [5]. HMGB1 is frequently overexpressed in cancer and has been reported to have both pro-tumoral and anti-tumoral effects [5]. HMGB1 can promote tumor cell survival, proliferation, invasion and metastasis, via the surface receptor for advanced glycation end-products (RAGE) in hepatocellular carcinoma [6, 7]. A pro-tumoral role of HMGB1 has also been identified in prostate cancer and endometrial cancer through the activation of Akt or Wnt signaling [8, 9]. Furthermore, HMGB1 can support angiogenesis and resistance to chemotherapy, and help maintain a chronic inflammatory tumor microenvironment that is beneficial to the tumor [10]. Conversely, the acute release of HMGB1 by dying tumor cells during chemotherapy and radiotherapy can induce anti-tumoral responses via TLR4 through immunogenic cell death (ICD) [11]. In some conditions, extracellular HMGB1 can also promote immune tolerance through regulatory T cells and myeloid-derived suppressor cells (MDSC) [12, 13]. The oxidized form of HMGB1 is highly present in the tumor microenvironment and can induce tolerogenic dendritic cells [14]. In addition, several studies showed that HMGB1 can polarize macrophages toward a pro-tumoral phenotype [15, 16]. Importantly, recent studies suggested that decreasing expression of HMGB1 in cancer cells can limit cancer progression and strengthen the antitumor immune response [7, 14, 16, 17]. However, the impact of tumor cell-intrinsic HMGB1 on the interplay between cancer and immune cells remains complex and more investigations are required before these insights may lead to therapeutic applications.

In this study we investigated the role of cancer cell-intrinsic HMGB1 expression on anti-tumor immunity in mice to expand the current knowledge in this field. We show that a decrease in HMGB1 expression in the murine CT26 colon cancer model leads to a reduction in tumor growth that is mediated by an enhanced antitumor immune response. We further show that this is linked to the local tumor microenvironment and is not driven by systemic anticancer immunity. Overall, we propose that cancer cell-intrinsic HMGB1 modulates the tumor microenvironment by preventing the recruitment and activation of immune effector cells into the tumor.

## Materials and methods

### Cell lines

All cell lines were obtained from ATCC. RAW264.7, J774, NIH3T3, B16-F10, MC38 and Hepa1-6 cells were cultured in Dulbecco’s modified Eagle’s medium (DMEM) (Gibco, Thermo Fisher) supplemented with 10% FBS (Biowest), 2mM L-glutamine (Gibco, Thermo Fisher) and 50 U/mL penicillin-streptomycin antibiotics cocktail (Gibco, Thermo Fisher). CT26, Renca and 4T1 cells were cultured in Roswell Park Memorial Institute-1640 (RPMI-1640) (Gibco, Thermo Fisher) medium supplemented with 10% FCS (Biowest), 2 mM L-glutamine (Corning, Fisher Scientific), 10 mM HEPES (Gibco, Thermo Fisher), 1 mM sodium pyruvate (Gibco, Thermo Fisher) and 50 U/mL penicillin-streptomycin antibiotic cocktail (Gibco, Thermo Fisher). Renca H2-Kb GFP cells were previously generated in our lab as described [18] and were cultured in RPMI 1640 (Gibco, Thermo Fisher) supplemented with 10% FBS (Biowest), 100 U/mL penicillin/streptomycin, 2 mM l-glutamine, 1 mM sodium pyruvate, and 1× minimum essential medium nonessential amino acids (Gibco, Thermo Fisher). All cells were maintained in a 37 °C humidified incubator with 5% CO_2_ and regularly tested negative for Mycoplasma species by PCR.

### Lentiviral transduction of tumor cells for shRNA-mediated downregulation

HMGB1 expression was knocked down in CT26 and B16-F10 cell lines using a lentiviral shRNA vector constructed in pLKO.1 (Sigma Aldrich). Lentiviruses were produced by transfecting HEK-293T cells with the indicated expression plasmids, together with pMD2.G envelope and psPAX2 packaging vectors (Addgene) using Lipofectamine 2000 transfection reagent (Invitrogen). For lentiviral transduction, cells were incubated with medium containing virus for 24 h. Cells were allowed to recover for 48 h before antibiotic selection with puromycin (Invivogen).

### Confocal laser scanning microscopy

To assess HMGB1 intracellular protein expression, HMGB1^mock^ and HMGB1^low^ CT26 cells were seeded on untreated German glass coverslips (6 mm in thickness and 12 mm in diameter, Karl Hecht GmbH & Co. KG “Assistent”, Sondheim/Rhön, Germany), which had previously been washed with acetone, ethanol, and ddH2O and baked in an oven. After 16 h, attached cells were washed with PBS pH 7.4, fixed with 4% paraformaldehyde (ThermoFisher Scientific) for 10 min, followed by permeabilization with 0.5% Triton X-100 (Calbiochem, Merck Millipore) in PBS pH 7.4 for 10 min, and blocked in blocking buffer (7.5% BSA, 30% normal goat sera (Santa Cruz Biotechnology), in PBS pH 7.4) for 1 h at room temperature. Rabbit anti-HMGB1 antibody (clone EPR3507, ab79823, Abcam) was diluted 1:250 in blocking buffer and cells were stained overnight at 4 °C. Cells were washed three times in PBS pH 7.4 with 0.025% Triton X-100, and then further incubated with the secondary antibody Alexa-568 goat anti–rabbit IgG (1:400 in 7.5% BSA with 0.025% Triton X-100; ThermoFisher Scientific) for 1 h at room temperature. Samples were extensively washed with PBS pH 7.4 with 0.025% Triton X-100, and F-actin was stained with 1.25% Alexa Fluor 488 Phalloidin (ThermoFisher Scientific) for 15 min in dark at room temperature. After washing with PBS, nuclei were counterstained with 1 µg/ml Hoechst 33,342 (ThermoFisher Scientific) for 10 min at room temperature in the dark. Cells were subsequently washed three times in PBS pH 7.4, and mounted in ProLong Gold mounting medium (ThermoFisher Scientific). Slides were examined, and images were acquired by an LSM 800 (Carl Zeiss Micro Imaging, Jena, Germany) with a 63×/1.40 oil differential interference contrast objective, followed by analysis with IMARIS software as previously reported [19].

### HMGB1 ELISA

HMGB1^mock^ and HMGB1^low^ CT26 cells were seeded four days prior to sample collection at a density of 1 × 10⁶ cells per T75 flask. At harvest, cell culture supernatants were collected and stored at − 20 °C until ELISA analysis. Cells were detached, centrifuged at 500 × *g* for 5 min, and counted. Cell pellets corresponding to 6 × 10⁶ cells were lysed in 200 µL of RIPA buffer (50mM TRIS pH 7.4, 150 mM NaCl, 1% NP-40, 1 mM EGTA, 1 µM NaF) supplemented with protease inhibitor cocktail (ratio 1:100, Sigma). Cells were sonicated on ice three times for 10 s with cooling intervals between cycles. Samples were then centrifuged at 18,000 × *g* for 15 min at 4 °C, and the clarified supernatants (= lysed cell material) were collected into clean microcentrifuge tubes and stored at − 20 °C until ELISA analysis. Concentration of HMGB1 in cell culture supernatants and lysed cell materials was measured by ELISA assay following manufacturer’s recommendations (Arigo Biolaboratories, ARG81310).

### *In vitro* viability assay

The cell counting kit-8 (CCK-8) (Sigma Aldrich) was used to assess in vitro cell viability of HMGB1^mock^ and HMGB1^low^ cells. 1 × 10^5^ cells/well were plated in 96-well plates containing complete medium and cells were allowed to adhere overnight. The next day, wells were washed and 100 µl of 1:10 diluted (in complete media) CCK-8 reagent was added in each well. The cells were incubated for 2 h in an incubator at 37 °C with 5% CO_2_. The absorbance was measured at 450 nm using a Clariostar Plus plate reader (BMG Labtech).

### *In vitro* BrdU incorporation assay

To determine the effect of HMGB1 downregulation in cancer cell proliferation a BrdU incorporation assay was used. For this, 1 × 10^5^ cells were seeded per well in a 96-well plate in complete medium and allowed to adhere overnight. Then, the cells were washed with sterile PBS and followed by BrdU assay according to the manufacturer’s instructions (Roche). Measurements were performed at 450 nm using a Clariostar Plus plate reader (BMG Labtech).

### Cell doubling time

Cells were thawed and maintained in culture, with passaging every 3–4 days (denoted as T, in days). At each passage, cells were counted both before splitting (denoted as Xb) and after trypsinization (denoted as Xe). The doubling time was calculated using the formula:

Doubling Time = [T × (ln2)] / [ln (Xe/Xb)]

Each round of passaging was plotted as an individual value to track the doubling time over successive passages.

### Live cell imaging

Cell proliferation was monitored in real-time using the Incucyte Live-Cell Imaging System. Cells were seeded in a 96-well plate at a density of 1000–5000 cells per well for B16-F10 cells and 2500–10,000 cells per well for CT26 cells and allowed to adhere overnight in a humidified incubator at 37 °C with 5% CO2. The plate was then placed in the Incucyte system, and images were captured every 6 h for a total duration of 7 days. Cell proliferation was quantified by measuring confluence over time using the Incucyte’s integrated software (Sartorius). Growth rates were calculated using non-linear regression models (exponential plateau for CT26 cell line and logistic growth for B16-F10 cell line) on GraphPad Prism 9.0 (GraphPad Software Inc., USA).

### Protein isolation and western blot analysis

Proteins were isolated from cell pellets using Pierce RIPA buffer (Thermo Fischer Scientific) containing complete protease inhibitor cocktail (Roche) and PhosSTOP phosphatase inhibitor cocktail (Roche) according to the manufacturer’s instructions. The protein amounts were determined using DC protein assay kit (Bio-Rad) relative to a standard curve generated with bovine serum albumin. 30–50 µg of protein was loaded onto 10% SDS-polyacrylamide gels, followed by electrophoresis together with a PageRuler Prestained protein ladder (10–250 kDa) (Thermo Fischer Scientific), and a wet transfer onto nitrocellulose membrane (GE Healthcare). Membranes were blocked in 5% skimmed milk for 1 h. Primary antibodies against mouse HMGB1 (Abcam, ab18256) and β-actin (Cell Signaling Technology #3700) were diluted 1:1000, applied overnight at 4 °C and subsequently probed with fluorescently labelled secondary antibodies (goat-anti-rabbit DyLight 680 and goat-anti-mouse IRDye 800 CW) diluted 1:10000. Blots were visualized on an Odyssey CLx scanner using Image Studio software (Version 5.2) (Li-Cor).

### Quantitative real-time PCR analysis for gene expression

Expression of given genes was confirmed using real-time reverse transcription-PCR (RT-PCR). Total RNA was isolated by using Trizol (Thermo Fischer Scientific), and cDNA was synthesized from 1 µg of total RNA using High Capacity cDNA Reverse Transcription kit (Applied Biosystems). As a control, a mock batch of cDNA was made without adding reverse transcriptase. The PCR reactions were run on QuantStudio 5 system with PowerUp SYBR Green Master mix (Applied Biosystems) and analysed using QuantStudio Design and Analysis software (Applied Biosystems) (See Table S1 for list of primer pairs). Results were only included in the analysis if the reaction with mock cDNA yielded a background signal.

### Immunostaining

Freshly collected tumors were embedded in OCT compound (WWR Chemicals), snap-frozen and stored at -80 °C. Ten µm sections were prepared using a cryostat (CryoStar™ NX70, Thermo Fisher) and stored at -20 °C. Slides were fixed with 4% paraformaldehyde and stained with the appropriated antibody mix diluted in Tris-buffered saline (TBS)-0.01% Tween 20 with 10% goat serum. The antibodies used were: rat anti-mouse CD4 (Acris Antibodies, clone RM4-5), rat anti-mouse CD8a (Biolegend, clone 53 − 6.7), rat anti-mouse/human CD11b-FITC (Biolegend, clone M1/70), rat anti-mouse CD31- Alexa Fluor^®^ 488 (biolegend, MEC13.3). Primary antibodies were paired with a goat anti-rat-Cy3 secondary antibody. All cryosections were stained with DAPI and images acquired with the multi-fluorescence slide scanner Axio Scan.Z1 (Zeiss). Pictures were analyzed using the software QuPath.

### Mice, tumor engraftment, and *in vivo* treatments of tumor-bearing mice

C57BL/6, BALB/c, OT-I, and NOD *SCID* gamma (NSG; NOD. Cg-*Prkdc*^*scid*^ *IL2rg*^*tm1Wjl*^/SzJ) mice were purchased from Charles River Laboratories. All mice were maintained under specific-pathogen-free conditions at the University of Geneva and used at 6–10 weeks of age. All animal experiments were performed according to the Swiss federal regulations, and all procedures were approved by the veterinary authorities of Canton of Geneva (GE/80/19, GE182C) and Canton of Bern (BE127/2022, BE34/2023).

For tumor induction, C57BL/6 and BALB/c mice were inoculated in the flanks, either as a single engraftment or as co-engraftment as indicated in each experiment, with 2.5–10 × 10^5^ CT26 or 2 × 10^5^ B16-F10 tumor cells subcutaneously. Tumor size is expressed as the product of the perpendicular diameters (mm^2^). In all experiments, mice were euthanized when they reached one of the criteria set by the local regulatory agency and the last measured value was included in calculation of the mean for the remaining time points, and subcutaneous tumor samples were collected for flow cytometry analysis.

For immune cell depletion, mice were injected intraperitoneally with 250 µg of rat anti-mouse CD8a, CD4 or control IgG2b (clones 2.43, GK1.5 and LTF-2 respectively, BioXcell) or 50 µg of anti-asialo GM1 antibody (eBioscience, San Diego, CA) at days 10, 13, and 17 after tumor induction. Target cell depletion was confirmed by flow cytometry.

### Tumor digestion, cell isolation and flow cytometric analysis

At the end of each in vivo experiment, subcutaneous tumors were collected from the flanks of the mice, and approximately 0.3 g of tumor from each sample were chopped into very small pieces and digested in a tumor dissociation kit (Miltenyi) at 37 °C for 20 min, as described in the manufacturer’s instructions. The digested samples were then filtered through a 40 μm cell strainer and washed twice with PBS (Gibco, Thermo Fisher). Red blood cell (RBC) lysis was performed using Pharm Lyse lysis buffer (BD Biosciences) for 5 min with three times repetition, followed by two washes with PBS. Then, cells were washed with FACS buffer (0.5% BSA and 0.5 mM EDTA in PBS), blocked with FcBlock TruStain fcX- CD16/32 (1:100, Biolegend) and stained with fluorochrome-coupled antibodies (Table S2) and a live-dead staining (Zombie, Biolegend). MuLV gp70 peptide-MHC tetramers (PE) (MBL international) for the MHC class I-restricted immunodominant epitope of CT26 cells, were used to determine CD8^+^ T cells that were tumor-specific. Cell acquisition was performed using a Novocyte flow cytometer (ACEA Biosciences). Data were analysed using the software FlowJo v10. For cell sorting, cells were stained with anti-CD45 for leukocytes, anti-CD31 for endothelial cells, anti-CD29 and anti-CD90.2 for fibroblasts and anti-CD44 for the selection of tumor cells. FcBlock TruStain fcX- CD16/32 (1:100, Biolegend) was added into the mix. A live-dead cell marker DRAQ7 (Thermo Fisher) was included prior to cell acquisition. Cell sorting was performed at the flow cytometry facility of the University of Geneva using a FACSAria Fusion™ (BD Biosciences) cell sorter.

### RNA sequencing

RNA from freshly collected cell lines, ex vivo whole tumor samples, or flow cytometry-sorted tumor samples, were extracted using Trizol, following manufacturer’s instructions. RNA concentration was measured with a NanoDrop spectrophotometer (Thermo Fischer Scientific) and RNA integrity was assessed with a Bioanalyzer2000 (Agilent Technologies). The TruSeq mRNA stranded kit (Illumina) was used for the library prepared with 200 ng of total RNA as input. Library molarity and quality were further assessed with the Qubit fluorimeter (Life Technologies) and Tapestation (Agilent Technologies) using a DNA High sensitivity chip (Agilent technologies). The libraries were pooled at 2 nM and loaded for clustering on the lane of a Single-read Illumina Flow cell. Reads of 100 bases were generated using the TruSeq SBS chemistry on an Illumina HiSeq 4000 sequencer. The normalization and differential expression analysis were performed with the R/Bioconductor package edgeR v3.16.5, for the genes annotated in the reference genome. Counts were filtered and normalized to library size (sequencing depth) and RNA composition (dispersion). Each sample is scaled using the TMM (trimmed Mean of M-values) normalization method. Pairwise comparison of the libraries and calculation of differentially expressed genes were performed using EdgeR package of R with general linear model and quasilikelihood F-test. Library preparation, sequencing and raw data handling were performed at the iGE3 Genomics Platform of the University of Geneva (https://ige3.genomics.unige.ch) Heatmap analysis, Over-Representation Analysis (ORA), Gene-set enrichment analysis (GSEA) and were produced with the web-based bioinformatics package NetworkAnalyst based on the recommended protocols and the web-based bioinformatics suite MetaCore (Thomson Reuters, New York, NY) was used to perform gene enrichment analysis.

### *In vitro* CD8^+^ T cell proliferation assay

CD8^+^ T cells were isolated from the spleens of C57BL/6 mice using the CD8^+^ T Cell Isolation kit (Miltenyi Biotec), and purity was verified by flow cytometry. Isolated cells were labeled with CellTrace™ Violet Cell Proliferation Kit (Invitrogen) according to the manufacturer’s instructions. Labeled CD8⁺ T cells were seeded in 96-well plates at 1 × 10⁵ cells per well and stimulated with anti-CD3/CD28 Dynabeads™ at a 1:1 bead-to-cell ratio (Gibco, Thermo Fisher Scientific) in the presence of recombinant mouse IL-2 (30 U/mL; PeproTech). Cells were treated with increasing concentrations of recombinant mouse HMGB1 protein (100–500 ng/mL; AbClonal). After 72 h of incubation at 37 °C, 5% CO₂, cells were analyzed by flow cytometry. Briefly, cells were washed with FACS buffer (PBS supplemented with 0.5% BSA and 0.5 mM EDTA), blocked with FcBlock™ TruStain fcX (anti-CD16/32; 1:100; BioLegend), and stained with fluorochrome-conjugated antibodies (Table S2) together with a viability dye (Zombie Green™; BioLegend). Data were acquired on a BD FACSLyric™ flow cytometer (BD Biosciences) and analyzed using FlowJo v10 software.

### *In vitro* cytotoxicity assay

Renca H2-K^b^ GFP cancer cells were seeded in 96-well plates at 1 × 10³ cells per well and allowed to adhere overnight. The following day, tumor cells were pulsed with the MHC class I–restricted, ovalbumin-derived SIINFEKL peptide (2 µg/mL; InvivoGen) for 4 h at 37 °C. CD8^+^ T cells were isolated from the spleens of OT-I mice using CD8^+^ T Cell Isolation kit (Miltenyi Biotec), and purity was verified by flow cytometry as described above. CD8⁺ T cells were then added to the Renca cultures at 1 × 10⁴ cells per well and treated with increasing concentrations of recombinant mouse HMGB1 protein (100–500 ng/mL; AbClonal). Co-cultures were maintained for 96 h at 37 °C, 5% CO₂. Tumor cell proliferation and survival were monitored longitudinally using the IncuCyte^®^ live-cell imaging system (Sartorius). Cell culture supernatants were collected, and IFN-γ concentrations were quantified by ELISA (BioLegend).

### Statistical analysis

All experiments were carried out as 2 or 3 independent biological replicates, each with at least 3 technical replicates, unless otherwise stated in the figure legends. GraphPad Prism 9.0 (GraphPad Software Inc., USA) was used for statistical data analysis. Unpaired two-tailed T test for comparison between two groups, paired T test for comparison of different variables on the same subject, one-way or two-way ANOVA followed by Tukey’s or Dunnet’s multiple comparison test for comparison between multiple groups were employed to determine the statistical difference. Heatmaps and volcano plots were generated using the web-based bioinformatics package NetworkAnalyst (http://www.networkanalyst.ca*)* following Ward’s hierarchical clustering and Euclidean distancing methods.

## Results

### HMGB1 downregulation in CT26 and B16-F10 cancer cell lines does not impact cell viability and proliferation *in vitro*

In order to select a suitable experimental system to test whether cancer cell-intrinsic HMGB1 contributes to antitumor immune responses, we generated murine cancer cell lines expressing different levels of HMGB1. All cancer cell lines available in our laboratory expressed HMGB1, albeit at different levels and variants (Fig. S1A), as previously described [20]. We selected CT26 murine colon adenocarcinoma cells, a cell line that is considered highly immunogenic, and B16-F10 murine melanoma cells, a cell line that is considered poorly immunogenic, for our experimental purposes [21]. Attempts to overexpress HMGB1 by lentiviral approaches in these cell lines were not successful; while the intrinsic expression was preserved, no ectopically expressed transcript could be detected, possibly due to post-translational degradation [22]. To downregulate HMGB1 expression, an shRNA-mediated approach was followed instead of a complete knockout of HMGB1, as the expression of HMGB1 in the nucleus is important for cell survival. The downregulation was successful in both cell lines, in which the expression of HMGB1 was significantly reduced both at protein and mRNA levels (Fig. S1B and C) and did not impact cell viability (Fig. S1D). Cellular localization of HMGB1 was restricted to the nucleus in both HMGB1^mock^ and HMGB1^low^ CT26 cells, as confirmed by confocal microscopy (Fig. S1E). Neither of the cell lines showed HMGB1 release under homeostatic conditions, but the intracellular content was downregulated in HMGB1^low^ cells (Fig. S1F). As HMGB1 is an important molecule for cell survival, HMGB1^low^ cell lines were further characterized for their proliferation properties. The downregulation of HMGB1 in CT26 and B16-F10 cancer cell lines did not impact their proliferation as tested by assessment of doubling time, BrdU incorporation, and confluence measured in real time (Fig. S2A-D). Furthermore, the downregulation of HMGB1 did not lead to any visible changes in the morphology of CT26 and B16-F10 cells (Fig. S2E). Overall, we showed that HMGB1 downregulation in CT26 and B16-F10 cancer cell lines did not result in any change in their morphology, viability and proliferation *in vitro.*

### Downregulation of tumor-intrinsic HMGB1 enhances immune cell recruitment to the tumor and prevents tumor growth

**Fig. 1:**
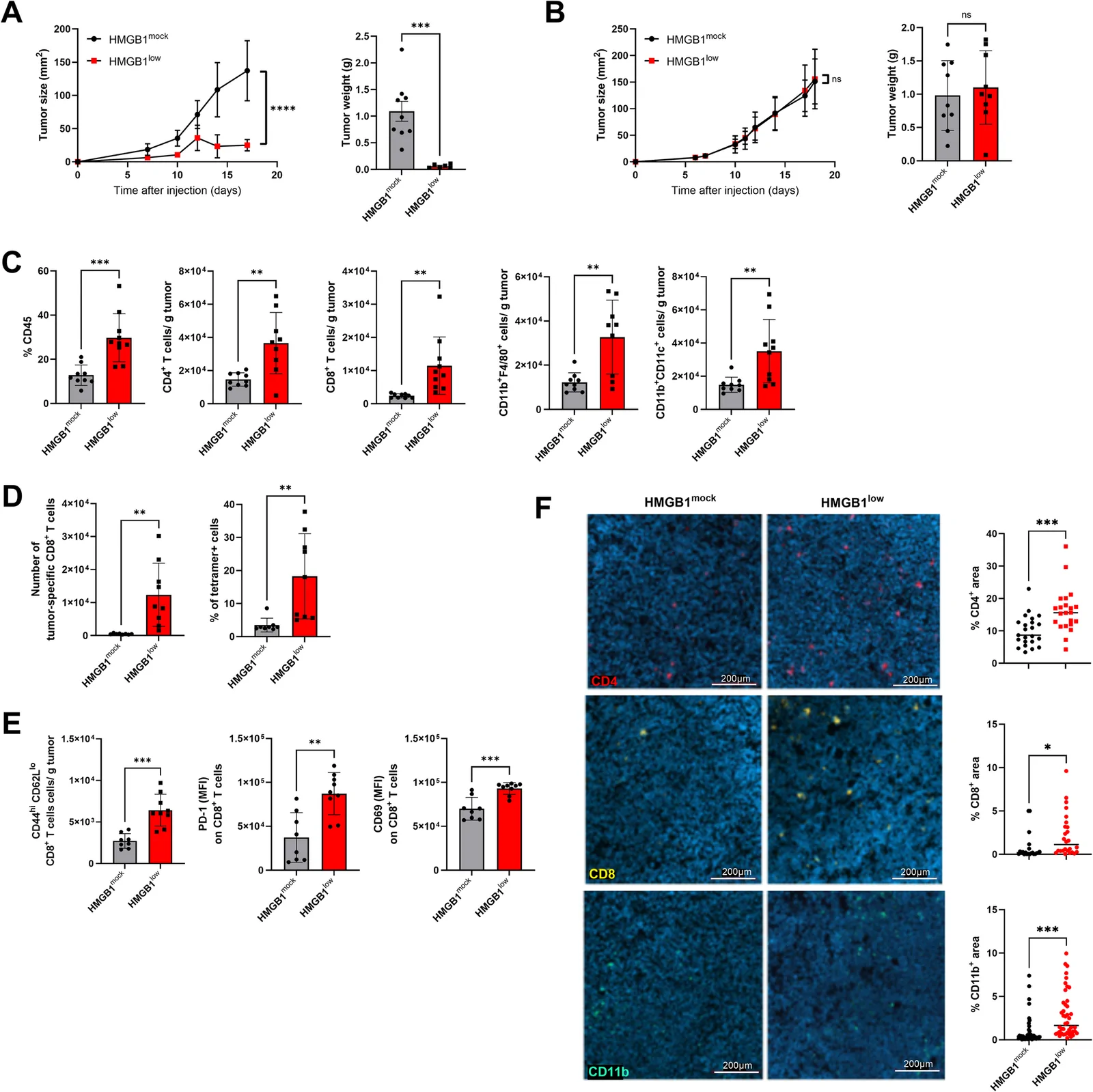
CT26 tumors, but not B16-F10 tumors, with low HMGB1 expression demonstrate reduced tumor growth. (**A**) Growth curve and endpoint weights of CT26 HMGB1^mock^ and HMGB1^low^ tumors. (**B**) Growth curve and endpoint weights of B16-F10 HMGB1^mock^ and HMGB1^low^ tumors. (**C**) Frequency of CD45^+^ immune cells among live cells and absolute numbers of CD4^+^ T cells, CD8^+^ T cells, CD11b^+^F4/80^+^ macrophages and CD11b^+^CD11c^+^ dendritic cells (DCs) per gram of CT26 tumor tissue measured by flow cytometry. (**D**) Number and frequency of tumour-infiltrating CD8^+^ T cells specific for the MHC-I-restricted immunodominant epitope of CT26 tumors (gp70 env). (**E**) Number of antigen-experienced CD44^hi^CD62L^lo^ CD8^+^ T cells per gram of CT26 tumor tissue and mean fluorescence intensity (MFI) of PD-1 and CD69 on the surface of CD8^+^ T cells. (**A-E**) Each dot indicates an individual mouse. Data are representative of three independent experiments. The graphs show the mean ± SD (*n* = 8–9 mice per group per experiment). (**F**) Representative images of immunostaining of CT26 HMGB1^mock^ and HMGB1^low^ tumors. CD4^+^, CD8^+^, and CD11b^+^ cells are labelled in red, orange, and green respectively. Nuclei are counterstained with DAPI. Quantification of immunostainings shown on the right. Marker-positive areas were quantified and expressed as percentage of ROI area (*n* = 3 mice/group, number of ROIs per biological sample = 7–17). Plots show median (black line) and individual values, each dot is one ROI. Paired Student’s t-test was applied for statistical analysis. ns: not significant, *p < 0.05, **p < 0.01, ***p < 0.001, ****p < 0.0001

We next investigated the contribution of cancer cell-intrinsic HMGB1 on tumor growth in mice. To achieve this, we injected HMGB1^mock^ and HMGB1^low^ CT26 or B16-F10 cells subcutaneously (s.c.) in BALB/c and C57BL/6 mice, respectively, and monitored the tumor growth. We found that the downregulation of HMGB1 led to a strong reduction in growth of CT26 tumors (Fig. 1A), although it did not affect the growth rate or viability of the cell line in vitro. This reduction in tumor growth was also observed in mice injected with two other CT26 clones with downregulation of HMGB1, but not with mock-transduced cell lines, nor with a cell line that had reverted to wild-type levels of HMGB1 after shRNA-mediated downregulation (line B12.2) (Fig. S3). In contrast, the downregulation of HMGB1 did not lead to a change in the growth of B16-F10 tumors (Fig. 1B). We then analysed the immune infiltrates associated with HMGB1^mock^ and HMGB1^low^ CT26 and B16-F10 tumors 14–18 days after the tumor cell injection. Not only was the frequency of CD45^+^ leukocytes higher in CT26 HMGB1^low^ tumors, but also the numbers of CD4^+^ and CD8^+^ T cells within this CD45^+^ leukocyte population were significantly higher in comparison to the HMGB1^mock^ control tumors (Fig. 1C and F). Similarly, the number of myeloid cells such as CD11b^+^ F4/80^+^ macrophages and CD11b^+^ CD11c^+^ dendritic cells (DCs) was higher in HMGB1^low^ tumors than in HMGB1^mock^ control tumors (Fig. 1C and F). To address whether the tumor-specific CD8^+^ T cell response was influenced by the downregulation of HMGB1 in cancer cells, we assessed the number of CD8^+^ T cells that specifically recognized the MHC-I restricted, immunodominant CT26 tumor antigen gp70 (SPSYVYHQF) in HMGB1^mock^ and HMGB1^low^ tumors. We observed that HMGB1^low^ tumors were infiltrated with higher numbers of tumor-specific CD8^+^ T cells than HMGB1^mock^ tumors (Fig. 1D). Additionally, more antigen-experienced (CD44^hi^ CD62L^lo^) CD8^+^ T cells were found in HMGB1^low^ tumors, and they exhibited higher levels of the activation markers PD-1 and CD69 on their surface (Fig. 1E). Thus, an enhanced immune infiltration was observed in HMGB1^low^ tumors in comparison to their mock controls, with higher numbers and higher activation levels of tumor-specific CD8^+^ T cells. In contrast, no significant change was seen in the CD45^+^ immune infiltrates of B16-F10 HMGB1^low^ tumors, including T cells, macrophages and dendritic cells (Fig. S4). Thus, we have shown that the downregulation of intracellular HMGB1 in cancer cells blocks the progression of CT26 tumors, enhances the accumulation of immune cells in the tumor and promotes the generation of tumor-specific CD8^+^ T cells. In B16-F10 tumors, which are generally considered to be poorly immunogenic [21], we did not observe any difference in tumor growth, nor in immune cell infiltration between HMGB1^low^ and HMGB1^mock^ tumors.

As direct contact through cell surface molecules plays an important role in the interactions between tumor cells and immune cells, we examined the expression of the key surface molecules PD-L1, MHC-I, CD80, and CD86 in HMGB1^mock^ and HMGB1^low^ CT26 and B16-F10 cancer cell lines by flow cytometry. We also immunophenotyped these cells following IFN-γ stimulation to mimic a situation where IFN-γ-secreting immune cells are present in the TME. Overall, the expression of these surface molecules was modestly altered in HMGB1^low^ cells compared with HMGB1^mock^ controls, with MHC class I and CD80 tending to be downregulated and CD86 upregulated (Fig. S5). Importantly, no major differences were observed between CT26 and B16-F10 cell lines, with the exception of PD-L1 (CD274), which was downregulated in HMGB1^low^ CT26 cells but remained unchanged in HMGB1^low^ B16-F10 cells. Based on these observations, we propose that although HMGB1 can modulate the expression of selected tumor cell surface molecules in both CT26 and B16-F10 models, these changes alone are unlikely to be the primary determinants of the divergent antitumor immune responses observed in vivo.

### The control in tumor growth following HMGB1 downregulation is due to a CD8^+^T cell immune response

**Fig. 2:**
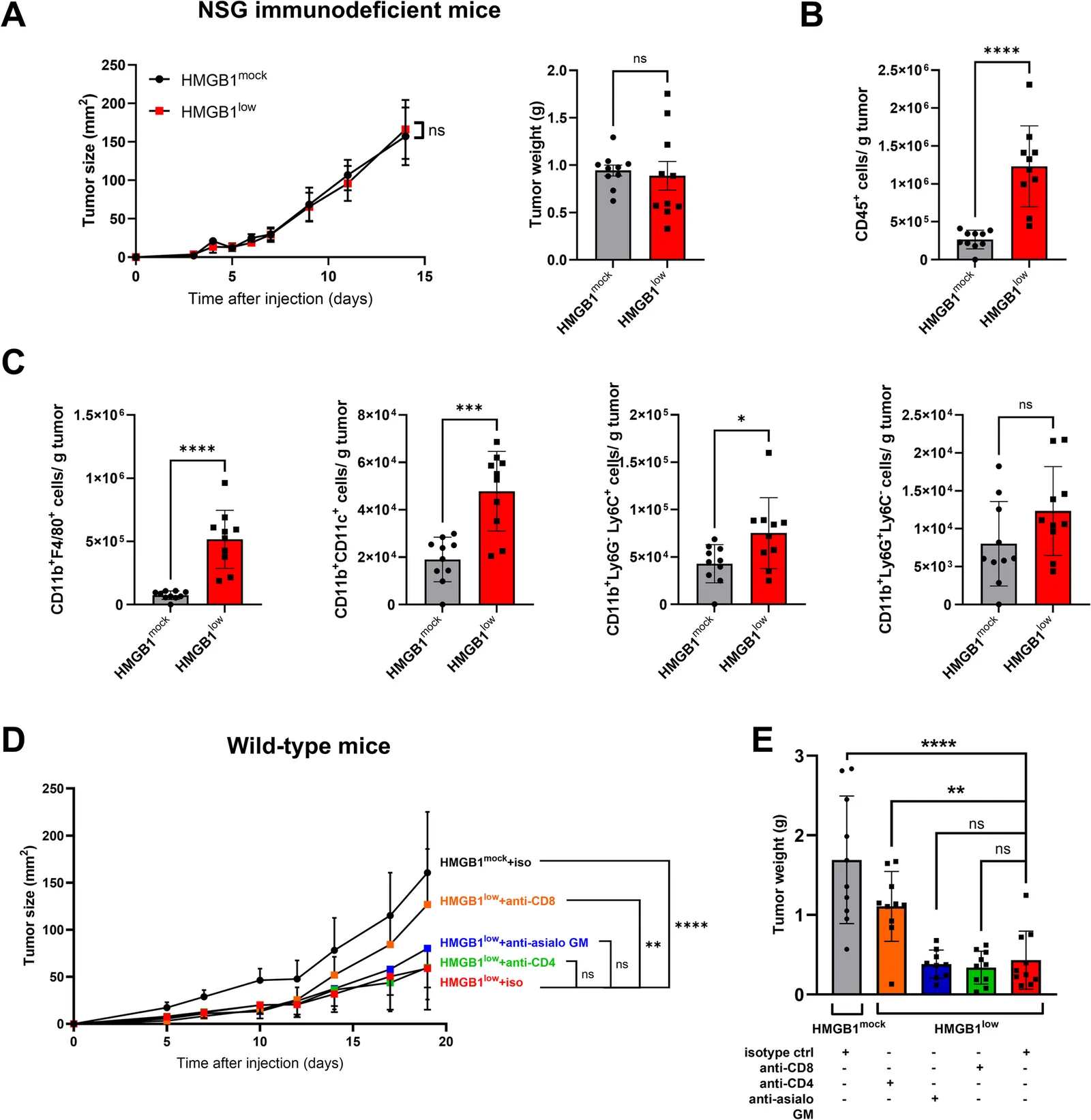
HMGB1 downregulation supports a CD8^+^ T cell-dependent anti-tumor immune response. (**A**) Growth curve and endpoint tumor weights of CT26 HMGB1^mock^ and HMGB1^low^ tumors that were inoculated subcutaneously into NSG mice. (**B**) Number of CD45^+^ immune cells analysed by flow cytometry at the endpoint. (**C**) Absolute numbers of CD11b^+^ F4/80^+^ macrophages, CD11b^+^CD11c^+^ DCs, CD11b^+^Ly6G^−^Ly6C^+^ and CD11b^+^Ly6G^+^Ly6C^−^ myeloid cells, per gram of tumor tissue within the CD45^+^ leukocyte population. (**D**) Growth curve and (**E**) endpoint tumor weights of CT26 HMGB1^mock^ and HMGB1^low^ cells that were inoculated subcutaneously into wild type BALB/c mice after depletion of CD4^+^ T cells, CD8^+^ T cells or NK cells by injection of the indicated antibodies. Data are represented as mean ± SD. Statistics were calculated on the individual days using the unpaired two-tailed Student’s t-test. Results are representative of two independent biological experiments for the NSG experiment, and three independent biological experiments for depletion (*n* = 10 per group per experiment). Paired Student’s t-test and one-way ANOVA was applied for statistical analysis. ns: not significant, *p < 0.05, p**<0.01, ***p < 0.001, ****p < 0.0001

We next investigated the contribution of the immune response in preventing the growth of HMGB1^low^ subcutaneous tumors. To observe this, we injected CT26 HMGB1^mock^ and HMGB1^low^ cells into NOD.Cg-*Prkdc*^*scid*^ *Il2rg*^*tm1Wjl*^/SzJ (NSG) mice that lack T, B and NK cells and cannot carry out proper cytokine signalling due to a null mutation in their *Il2rg* receptor. In these mice, both HMGB1^mock^ and HMGB1^low^ tumors showed similar growth, indicating that the decreased growth of HMGB1^low^ tumors in immunocompetent mice is due to an enhanced immune response (Fig. 2A). Interestingly, NSG mice still showed an increase in total infiltrating leukocytes in HMGB1^low^ tumors compared to HMGB1^mock^ tumors (Fig. 2B). Furthermore, in NSG mice the CD11b^+^F4/80^+^ macrophages, CD11b^+^CD11c^+^ DCs and CD11b^+^Ly6G^−^Ly6C^+^ myeloid cell numbers were significantly higher in HMGB1^low^ tumors (Fig. 2C), as seen in wild-type mice (Fig. 1C). These results suggests that the impact of HMGB1 downregulation on tumor growth is mediated by the immune compartment.

To elucidate the contribution of specific lymphocyte populations in controlling tumor growth, we depleted CD4^+^ T cells, CD8^+^ T cells or NK cells by use of depletion antibodies in wild-type mice bearing palpable HMGB1^low^ tumors. Successful depletion was verified by flow cytometry in the blood of treated mice (Fig. S7). While the depletion of CD4^+^ T cells and NK cells did not lead to any change in tumor progression, depletion of CD8^+^ T cells restored the growth and weight of HMGB1^low^ tumors to levels similar to HMGB1^mock^ tumors (Fig. 2D and E). Thus, we conclude that cancer cell-intrinsic downregulation of HMGB1 enhances an antitumor immune response and promotes infiltration by effector CD8^+^ T cells that results in the control of tumor growth.

**Fig. 3:**
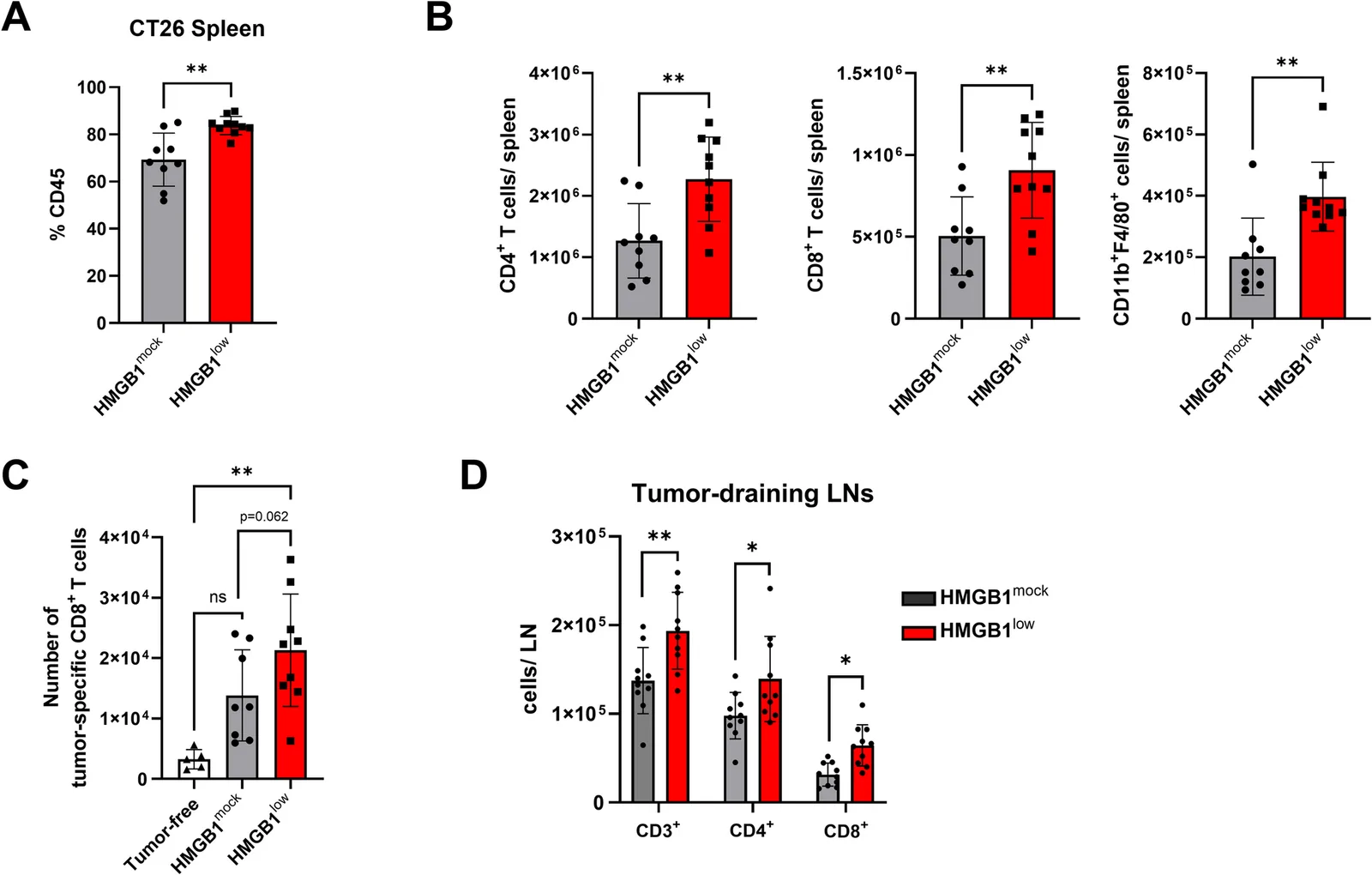
HMGB1^low^ tumors induce a stronger immune activation in secondary lymphoid organs. (**A**) Percentages of CD45^+^ immune cells in spleen of tumor-bearing mice analysed by flow cytometry at the endpoint. (**B**) Absolute numbers of CD4^+^ T cells, CD8^+^ T cells and CD11b^+^F4/80^+^ macrophages per spleen. (**C**) Number of tumor-specific CD8^+^ T cells per spleen. (**D**) Number of CD3^+^, CD4^+^ and CD8^+^ T cells in the tumor-draining lymph nodes (LNs) of HMGB1^mock^ and HMGB1^low^ tumor-bearing mice. Each dot represents one individual mouse. The graphs show the mean ± SD. Student’s t-test and one-way ANOVA was applied for statistical analysis. ns: not significant, *p < 0.05, **p < 0.01

To examine whether cancer cell-intrinsic HMGB1 impacts the immune landscape, we examined the immune composition of spleens and tumor-draining lymph nodes from mice bearing HMGB1^mock^ or HMGB1^low^ CT26 tumors. Importantly, the increase in immune cell infiltration in the tumors was associated with an increase in leukocyte numbers in the spleen of HMGB1^low^ tumor-bearing mice (Fig. 3A). Similarly, numbers of CD4^+^ and CD8^+^ T cells, as well as CD11b^+^ F4/80^+^ macrophages, were significantly higher in the spleens of these mice (Fig. 3B). We also assessed the number of splenic CD8^+^ T cells that specifically recognized the immunodominant peptide of CT26 tumors in HMGB1^mock^ and HMGB1^low^ tumor-bearing mice, as well as in tumor-free mice. While both tumor-bearing groups showed increased numbers of tumor-specific CD8^+^ T cells in the spleen in comparison to tumor-free mice, we observed much higher numbers of these cells in the spleens of HMGB1^low^ tumor-bearing mice (Fig. 3C). These findings were also accompanied with increased leukocyte numbers in tumor-draining lymph nodes. Indeed, the tumor-draining lymph nodes of mice bearing HMGB1^low^ tumors exhibit higher numbers of CD3^+^, CD4^+^ and CD8^+^ T cells (Fig. 3D). Overall, these results indicate that HMGB1^low^ tumors lead to modifications in the immune landscape compared to HMGB1^mock^ tumors, with elevated T cell numbers in the secondary lymphoid organs.

### The downregulation of cancer cell-intrinsic HMGB1 strongly impacts gene expression in the tumor microenvironment

**Fig. 4:**
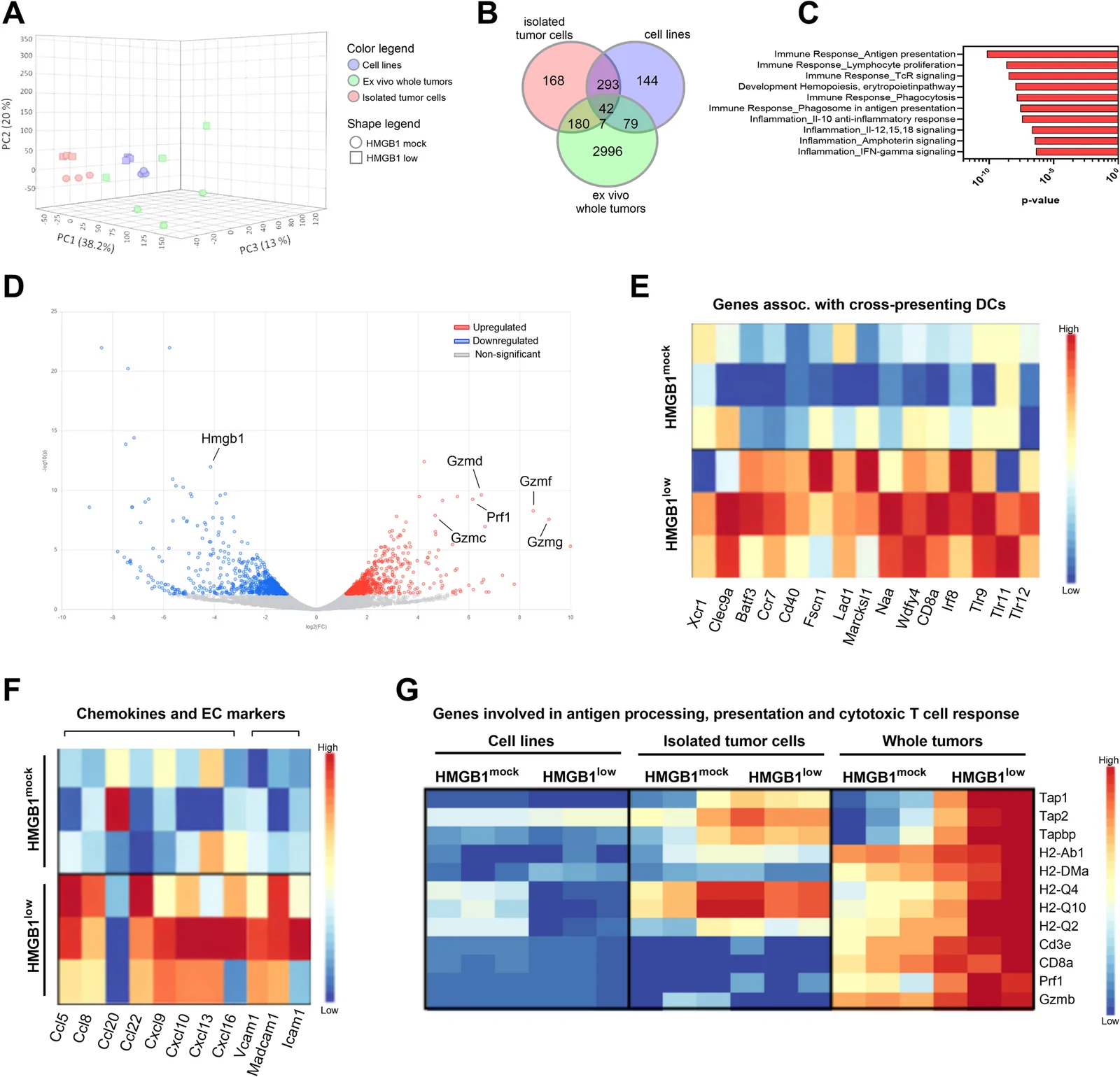
Gene expression and immune pathways are altered upon HMGB1 downregulation in CT26 tumors. (**A**) 3D principal component analysis (PCA) clustering of RNA-seq gene expression data from CT26 HMGB1^mock^ and HMGB1^low^ cell lines, ex vivo whole tumors and isolated tumor cells (*n* = 3 per group). (**B**) Venn diagram showing number of differentially expressed genes in each comparison. (**C**) Pathway analysis of MetaCore Process Networks for differentially expressed genes in HMGB1^low^ tumors compared to HMGB1^mock^ tumors (top 10 pathways are shown). (**D**) Volcano plot of differentially expressed genes between HMGB1^mock^ and HMGB1^low^ whole tumors (cut-off of 2-fold changes with 5% false discovery rate (FDR)). Every point represents one gene plotted according to the fold change (Log_2_FC) and p-value (-Log_10_P-value). Red dots specify upregulated, blue dots specify downregulated genes in HMGB1^low^ tumors. (**E**-**G**) Endpoint tumors were collected from HMGB1^mock^ and HMGB1^low^ tumors and bulk RNA was extracted from whole tumors for RNA sequencing. Heatmaps display differentially expressed genes associated with (**E**) cross-presenting DCs, (**F**) leukocyte-attracting chemokines and adhesion molecule markers that can be expressed on endothelial cells (EC) and (**G**) genes that are involved in antigen processing, presentation and cytotoxic T cell responses

In order to better characterize the effect of HMGB1 downregulation in tumors in a non-biased approach, we performed transcriptional profiling for HMGB1^mock^ and HMGB1^low^ whole tumors and for the cultured cell lines. We also included an analysis of whole tumors from which leukocytes, endothelial cells and fibroblasts were removed by fluorescent-activated cell sorting (isolated tumor cells). In a 3D principal component analysis (PCA) plot, expression profiles from HMGB1^low^ tumors showed a distinct clustering compared to the HMGB1^mock^ tumors, which was the case also for HMGB1^mock^ vs. HMGB1^low^ cell lines and isolated tumor cells (Fig. 4A). We found 144 differentially expressed genes (DEGs) in HMGB1^mock^ vs. HMGB1^low^ cell lines, 168 DEGs in isolated tumor cells, and 2996 DEGs in whole tumors (Fig. 4B). In a MetaCore process network analysis, we observed differential expression of genes involved in immune response and inflammatory pathways, such as antigen presentation, lymphocyte proliferation and TcR signaling between HMGB1^mock^ and HMGB1^low^ whole tumors (Fig. 4C). Of note, in the list of differentially expressed pathways we found amphoterin (HMGB1) signaling, which highlights the involvement of HMGB1 in this context (Fig. 4C). In HMGB1^low^ whole tumor samples, we observed upregulation of genes that are associated with immune cytolytic activities including granzymes (*Gzmc*, *Gzmd*, *Gzme*, *Gzmf*, and *Gzmg*), perforin (*Prf1*) and interferon-γ (*Ifng*), and as expected downregulation of *Hmgb1* (Fig. 4D). Genes associated with interferon-α/β responses tended to be upregulated in HMGB1^low^ tumors, suggesting that enhanced type I interferon signaling may contribute to the increased T-cell response (data not shown). Notably, HMGB1^low^ tumors also showed upregulation of distinctive markers of tumor-infiltrating cross-presenting DCs (Fig. 4E), which can promote the proliferation and effector functions of intratumoral T cells [23, 24]. At the same time, we show that transcription of chemokines and vascular adhesion molecules that enhance T cell infiltration is upregulated in HMGB1^low^ whole tumors (Fig. 4F). These data are further in line with over-representation analysis (ORA) of KEGG pathways, including, but not limited to, pathways in antigen processing and presentation, and cytotoxic T cell responses, suggesting that HMGB1^low^ tumors more effectively present endogenous antigens, are potentially better at forming immunological synapses in the tumor microenvironment and elicit high expression of cytotoxic molecules associated with cytotoxic T cells (Fig. 4G). All in all, the transcriptomic enrichment of genes and pathways supports the observation that HMGB1^low^ tumors are highly infiltrated with immune cells, and that cancer cell-intrinsic HMGB1 controls the local immune microenvironment resulting in limited anti-tumor immunity.

### Cancer cell-intrinsic downregulation of HMGB1 elicits a tumor-intrinsic immune response

Although HMGB1^low^ tumors induced a systemic tumor-specific response in secondary lymphoid organs, transcriptomic analysis of whole tumors suggested changes in the microenvironment that enhanced local immune infiltration. To determine whether these observations could be attributed to a direct effect of extracellular HMGB1 on CD8⁺ T cells, we performed in vitro experiments comparing CD8⁺ T-cell activation and effector function in the presence or absence of recombinant HMGB1 (Fig. S8). Recombinant HMGB1 did not affect the expression of activation markers, IFN-γ production, proliferative capacity, or antigen-specific cytotoxic activity of CD8⁺ T cells. Notably, in our model HMGB1 was exclusively detected in the nucleus of cancer cells, with no evidence of cytosolic or extracellular localization (Fig. S1). Collectively, these results suggest that the immunomodulatory effects of cancer cell–intrinsic HMGB1 are more likely attributable to its nuclear functions rather than to extracellular HMGB1 signaling.

**Fig. 5:**
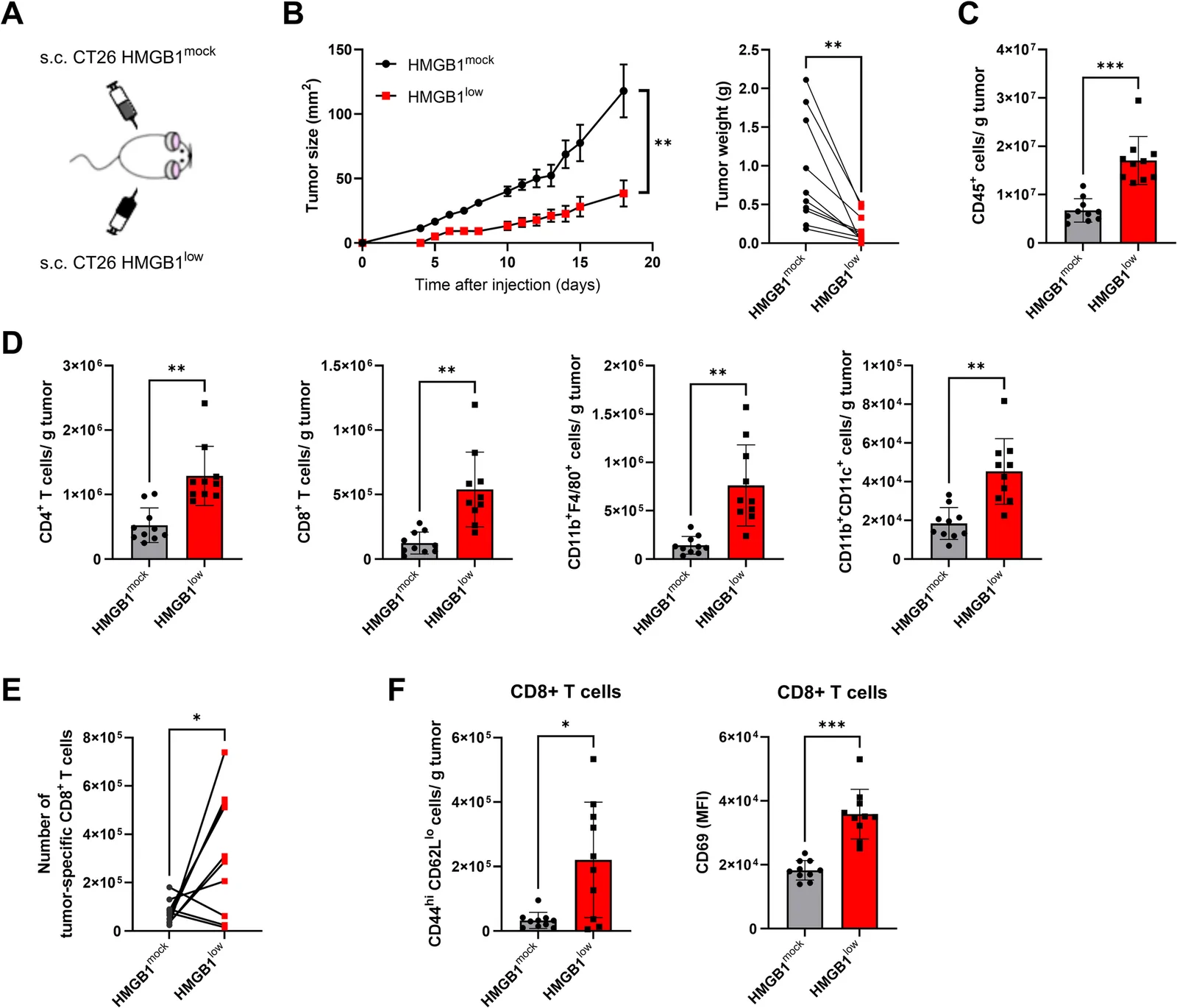
Anti-tumor immune responses in HMGB1^low^ tumors remain tumor-intrinsic. (**A**) Experimental design for B to F. CT26 HMGB1^mock^ and HMGB1^low^ cells were injected subcutaneously into flanks of BALB/c mice (*n* = 10 mice per group). (**B**) Growth curve and weights of bilaterally injected CT26 HMGB1^mock^ and HMGB1^low^ tumors. (**C**) Number of CD45^+^ immune cells per gram of tumor analysed by flow cytometry at the endpoint. (**D**) Absolute numbers of CD4^+^ T cells, CD8^+^ T cells, CD11b^+^F4/80^+^ macrophages and CD11b^+^CD11c^+^ DCs per gram of tumor tissue. (**E**) Number of tumor-specific CD8^+^ T cells in the tumor. (**F**) Number of antigen-experienced CD44^hi^CD62L^lo^ CD8^+^ T cells per gram of tissue and mean fluorescence intensity (MFI) of activation marker CD69 on CD8^+^ T cells. Results are representative of two independent biological experiments (*n* = 10 mice per group per experiment). Each dot represents one individual mouse. The graphs show the mean ± SD. Paired Student’s t-test and one-way ANOVA was applied for statistical analysis. *p < 0.05, **p < 0.01, ***p < 0.001

In order to investigate whether the immune response in HMGB1^low^ tumors could lead to immunity against distant tumors, we inoculated subcutaneous CT26 HMGB1^mock^ and HMGB1^low^ tumors in separate flanks of the same mice (Fig. 5A). Interestingly, although HMGB1^low^ tumor growth was controlled as seen in mice bearing single tumors, HMGB1^mock^ tumor growth was not reduced, implying that effects of HMGB1^low^ tumors remain tumor-intrinsic and fail to elicit abscopal immunity against distant HMGB1^mock^ tumors (Fig. 5B). The numbers of infiltrating leukocytes were higher in HMGB1^low^ tumors compared to their HMGB1^mock^ counterparts (Fig. 5C), which was accompanied by increased numbers of CD4^+^ and CD8^+^ T cells, CD11b^+^ F4/80^+^ macrophages, CD11b^+^ CD11c^+^ DCs (Fig. 5D), as well as higher numbers of tumor-specific and antigen-experienced CD8^+^ T cells in HMGB1^low^ tumors (Fig. 5E and F). Thus, the control of tumor growth and immune cell recruitment was restricted to HMGB1^low^ tumors. Overall, these results suggest that cancer cell-intrinsic HMGB1 plays a key role in the local regulation of the immune response by limiting the recruitment and activation of immune cells in the tumor microenvironment.

## Discussion

Extracellular HMGB1 is an indispensable component of the immune system that is involved in many inflammatory processes [25, 26]. Whether HMGB1 is passively released from injured cells or actively secreted from stimulated immune cells, it triggers inflammation by promoting the release of cytokines such as IL-6 and TNF-α [21] and inducing leukocyte chemotaxis [4, 27]. In cancer, HMGB1 mediates the immunogenic cell death (ICD) of tumor cells upon chemotherapy or radiotherapy and thereby potentiates the development of an antitumor immune response [11, 28]. In fact, dying tumor cells lacking HMGB1 are less potent in initiating an antitumor immune response after chemotherapy [29]. Despite these well-characterized proinflammatory functions of HMGB1, we have found that the downregulation of HMGB1 in CT26 tumor cells leads to an enhanced antitumor immune response in mice and a reduction in tumor growth, confirming and expanding on previous findings showing a protumoral effect of HMGB1 in murine cancer models [14–17]. Furthermore, we have clearly shown that the decrease in HMGB1^low^ tumor progression was due to the development of adaptive anticancer immunity, since this effect was abolished in immunodeficient NSG mice.

In HMGB1^low^ CT26 tumors, we observed enhanced recruitment of several different types of immune cells in immunocompetent mice, including CD4^+^ and CD8^+^ T cells, macrophages, and dendritic cells. By using depleting antibodies, we demonstrated that the limitation in HMGB1^low^ tumor growth is dependent on CD8^+^ T cells. We further showed that the number of antigen-experienced and activated CD8^+^ T cells was increased in HMGB1^low^ tumors compared to HMGB1^mock^ tumors, as was the number of CD8^+^ T cells that were specific for the immunodominant CT26 epitope. We thus show that HMGB1 is a tumor cell-intrinsic factor that suppresses the infiltration of immune effector cells into the tumor microenvironment and limits anti-tumor immunity.

Interestingly, in NSG mice that are devoid of mature lymphocytes, we still observed an increase in myeloid cells within HMGB1^low^ tumors, including macrophages, dendritic cells and CD11b^+^ Ly6G^−^ Ly6C^+^ monocytic cells. This suggests that the recruitment of myeloid cells into these tumors occurs independently of the immunostimulatory effects of IFN-γ produced by tumor-infiltrating lymphocytes [30]. Gene expression analysis revealed that antigen presentation, lymphocyte proliferation and TCR signaling were enhanced in HMGB1^low^ tumors, supporting the hypothesis that cancer cell-intrinsic HMGB1 prevents T-cell priming. In addition, in HMGB1^low^ tumors we observed upregulated expression of a panel of genes associated with a subset of cross-presenting dendritic cells that are known to foster local re-activation of CD8^+^ T cells [23]. Importantly, this DC subset is associated with improved overall survival and success of immunotherapy in patients. We indeed observed upregulation of genes coding for leukocyte-attracting chemokines and adhesion molecules in HMGB1^low^ tumors. These pathways were enhanced mainly in whole tumors but not in the cancer cell lines or in isolated tumor cells. This finding suggests that cancer cell-intrinsic HMGB1 shapes the tumor microenvironment and thereby limits the development of effective antitumor immunity. Our results are consistent with previous studies in other tumor models, including the MC38 colorectal cancer model and the 4T1 breast cancer model, which demonstrated immunosuppressive functions of HMGB1. In these settings, extracellular HMGB1 was shown to promote the recruitment and activation of immunosuppressive cell populations, such as regulatory T cells and myeloid-derived suppressor cells, and to impair the recruitment and function of effector T cells and dendritic cells [12–14, 17, 31]. In contrast, our findings reveal a previously unrecognized mechanism whereby tumor cell-intrinsic nuclear HMGB1 contributes to immune evasion, highlighting a distinct effect of HMGB1-mediated immunoregulation within the tumor microenvironment.

It is noteworthy that the effect of cancer cell-intrinsic HMGB1 on tumor growth was only observed in CT26 tumors, but not in B16-F10 tumors, even though both models express HMGB1 at high levels. Phenotypic characterization of the cell lines showed that although HMGB1^low^ tumors differentially expressed MHC-I and costimulatory molecules compared to their wild-type counterparts, this was seen in both CT26 and B16-F10 cell lines, suggesting that this was not the source of the difference in response to HMGB1 downregulation. Rather, we propose that this may be explained by the antigenicity and immunogenicity levels of the models. Among a set of syngeneic tumor models, the colorectal carcinoma model CT26 was shown to be the most immunogenic, whereas the B16-F10 melanoma was the least immunogenic, with CT26 also exhibiting a higher mutational burden that can provide more neoepitopes for T-cell recognition [32–34]. Others have reported an impact of cancer cell-intrinsic HMGB1 in B16-F10 tumors, a difference which may be attributable to the absence of complete knockout in the present study [17]. In addition, the functions of HGMB1 are modulated according to its redox state [35]. Recently, it has been shown that the oxidized form of HMGB1 had tolerogenic properties on dendritic cells, impeding the antitumor immune response [14]. Therefore, it is possible that the oxidation status of HMGB1 within the tumor may affect its impact on the antitumor response and lead to a context-dependent impact of HMGB1 downregulation. Notably, HMGB1 undergoes oxidization primarily following secretion or extracellular release, whereas nuclear HMGB1 remains fully reduced [36], suggesting that redox-dependent immunomodulatory functions are likely to be restricted to extracellular HMGB1.

It would be worthy to mention that downregulation of HMGB1 may have significant impacts on the induction of immunogenic cell death (ICD), in particular, during conventional cancer therapies such as chemotherapy and radiotherapy. HMGB1 acts as a damage-associated molecular pattern (DAMP) molecule, which is released from dying tumor cells and subsequently triggers an immune response [4]. When HMGB1 is downregulated, the release of DAMPs is diminished, leading to a reduced activation of the immune system. This attenuation can result in a lower recruitment and activation of dendritic cells and other antigen-presenting cells, which are crucial for the initiation of an adaptive immune response against tumor cells. Consequently, the effectiveness of chemotherapy and radiotherapy in promoting an anti-tumor immune response is compromised, potentially leading to decreased overall therapeutic efficacy. Therefore, maintaining adequate levels of extracellular HMGB1 would be critical for the optimal induction of ICD and the subsequent activation of an effective anti-tumor immune response during conventional cancer treatments.

Although mice with HMGB1^low^ CT26 tumors exhibited an elevated immune response in secondary lymphoid organs, there was no abscopal effect in mice injected bilaterally with an HMGB1^low^ and an HMGB1^mock^ tumor. Indeed, HMGB1^mock^ tumors in these mice did not show enhanced immune infiltration or the presence of antigen-experienced, activated CD8^+^ T cells. These findings indicate that the antitumoral immune effects associated with HMGB1 downregulation are largely confined to the local tumor microenvironment.

Protumoral and immunosuppressive functions of HMGB1 have previously been attributed to its cytoplasmic expression and active extracellular release, particularly in triple-negative breast cancer [14], and cytosolic HMGB1 has also been implicated in the induction of autophagy under cellular stress conditions [37]. In contrast, HMGB1 was exclusively localized to the nucleus in CT26 cancer cells, arguing against a major contribution of extracellular or cytosolic HMGB1-mediated immunosuppression in this model. Consistent with this interpretation, treatment with recombinant HMGB1 protein did not directly affect CD8⁺ T-cell activation, proliferation, or cytotoxic function in vitro, and pharmacological targeting of extracellular HMGB1 failed to confer therapeutic benefit in tumor-bearing mice (data not shown).

Together, these observations suggest that, beyond the previously described extracellular and cytosolic functions of HMGB1, cancer cell–intrinsic, nuclear HMGB1 modulates local tumor-intrinsic factors that are critical for shaping the antitumor immune response. By limiting the recruitment of tumor-reactive immune cells, HMGB1 promotes tumor growth and restrains the development of an effective local antitumor immune response. In this context, elevated levels of cancer cell–intrinsic, nuclear HMGB1 may represent a prognostic marker of immune evasion. Importantly, these findings also underscore the need for innovative therapeutic strategies capable of selectively targeting HMGB1 within this specific cellular compartment in order to effectively enhance antitumor immunity.

## Supplementary Information

Below is the link to the electronic supplementary material.

Supplementary Material 1 (PDF 2.79 MB)

## Data Availability

Data that support the findings of this study are available within the article and the supplementary materials.

## Abbreviations

HMGB1: High mobility group box protein 1
DAMP: Danger-associated molecular pattern
RAGE: Receptor for advanced glycation end-products
ICD: Immunogenic cell death
MDSC: myeloid-derived suppressor cells
ORA: Over-Representation Analysis
GSEA: Gene-set enrichment analysis
DEG: Differentially expressed gene
