# Supplementary material for: HMGB1 expression in CT26 murine cancer cells modulates the tumor microenvironment and limits local anti-tumor immunity
Source: Cell Mol Life Sci. 2026 Aug 4;83(1):403. doi: 10.1007/s00018-026-06334-7 (PMC13737301; doi:10.1007/s00018-026-06334-7)
Supplement: Supplementary file 1 [file 18_2026_6334_MOESM1_ESM.pdf]

HMGB1 expression in CT26 murine cancer cells modulates the tumor microenvironment and limits local anti-tumor immunity

Betül Taşkoparan[1,2], Viola Puddinu[1,2], Bart Boersma[1,2], Lorenzo Spagnuolo[1,2], Hélène Poinot[1,2], Eloise Dupuychaffray[1,2], Montserrat Alvarez[1,2], Muriel Urwyler[1,2], Marietta Margareta Korsos[3], Darko Stojkov[3], Aurélien Pommier[1,2], Paul R. Walker[4], Carole Bourquin[1,2,3,4,5*]

[1]School of Pharmaceutical Sciences, University of Geneva

[2]Institute of Pharmaceutical Sciences of Western Switzerland, University of Geneva

[3]Institute of Pharmacology, University of Bern, Bern, Switzerland.

[4]Translational Research Centre in Oncohaematology, Faculty of Medicine, University of Geneva, Geneva, Switzerland.

[5]Department of Anaesthetics, Pharmacology, Intensive Care and Emergencies, Faculty of Medicine, University of Geneva, Geneva, Switzerland

*corresponding author: carole.bourquin@unibe.ch

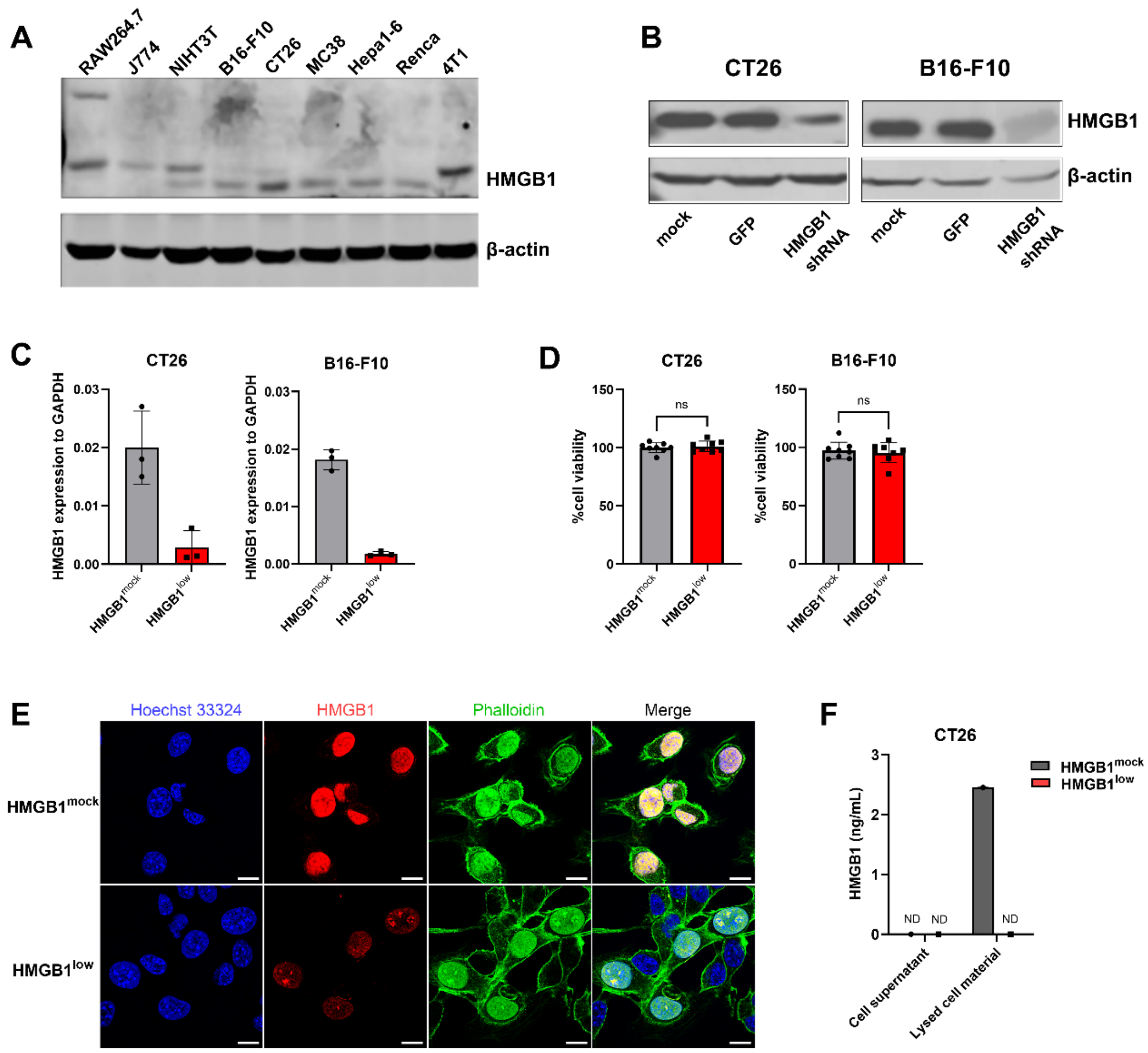

Fig. S1 HMGB1 downregulation in CT26 and B16-F10 cell lines does not impact cell viability and protein localization *in vitro.* (**A**) Immunoblot of lysates from RAW264.7, J774, B16-F10, CT26, MC38, Hepa1-6, Renca, 4T1 cells probed with HMGB1 (Mw:29kDa) and β-actin (Mw:42kDa) antibodies. (**B**) Immunoblot of lysates from CT26 and B16-F10 cell lines transduced with control shRNA (mock and GFP) and HMGB1 shRNA probed with HMGB1 and β-actin antibodies. (**C**) Relative mRNA expression of HMGB1 in CT26 and B16-F10 HMGB1^{mock} and HMGB1^{low} cell lines. (**D**) Percentage of viable cells of CT26 and B16-F10 HMGB1^{mock} and HMGB1^{low} cells measured by CCK-8 viability assay. (**C-D**) Each dot represents one biological replicate, and the graphs show the mean ± SD of independent experiments. ns: not significant (**E**) HMGB1 localization in HMGB1^{mock} and HMGB1^{low} CT26 cells assessed by confocal microscopy. HMGB1 was immunostained using an anti-HMGB1 antibody and a secondary anti-rabbit Alexa Fluor 568 antibody (red). Nuclei were stained with 33342 Hoechst (blue). Actin filaments associated with cell membrane were counterstained with phalloidin (green). In the merged images, HMGB1 is shown in yellowish color. Representative images of at least 3 independent experiments are shown. Scale bars: 10 µm. (**F**) Quantification of HMGB1 release by HMGB1^{mock} and HMGB1^{low} CT26 cells measured by ELISA. HMGB1 was measured in cell supernatants and lysed cell material. ND: not detected

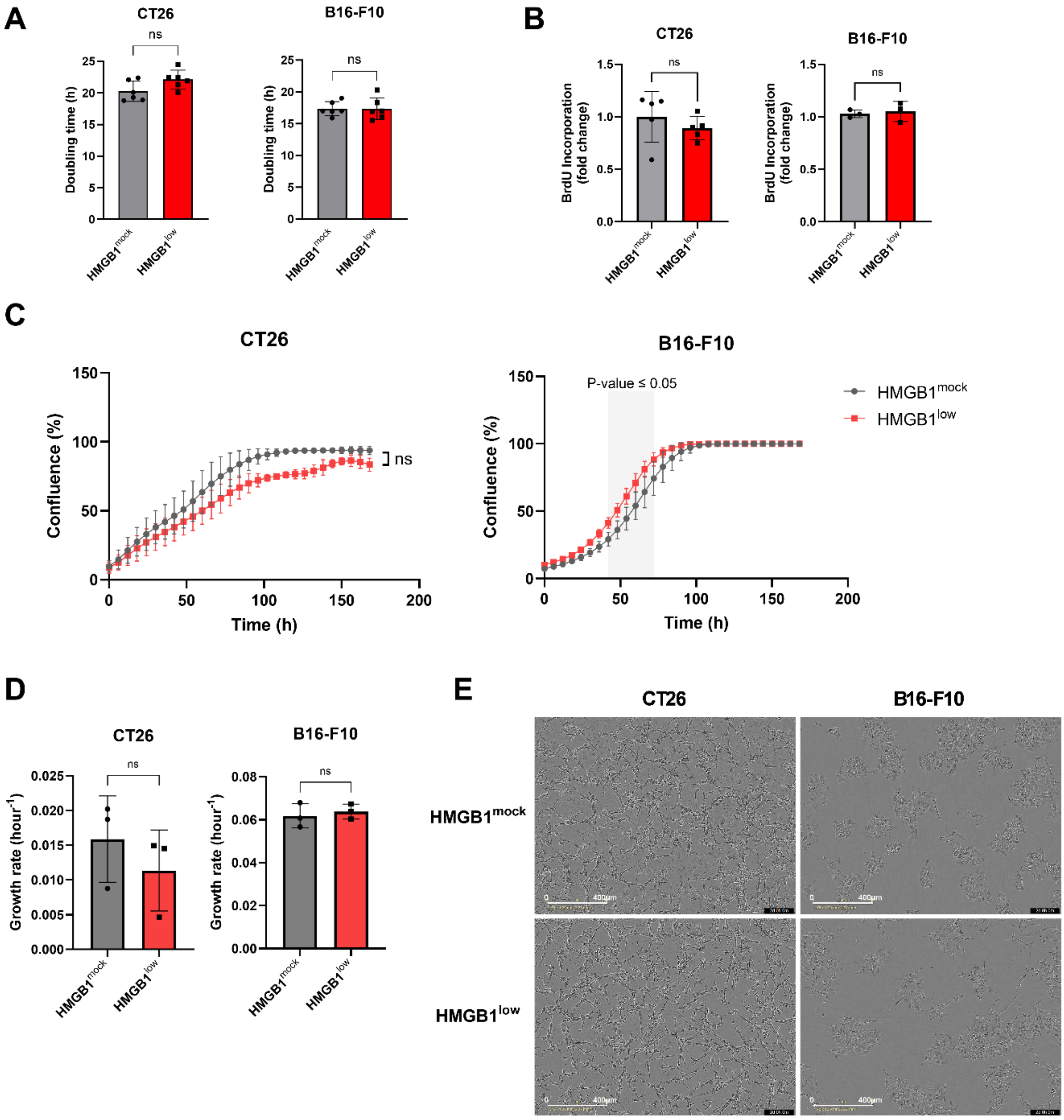

Fig. S2 Effect of HMGB1 knockdown on cell proliferation in vitro. (**A**) Quantification of cell doubling time of HMGB1^{mock} cells (gray bars) and HMGB1^{low} cells (red bars) in the B16-F10 and CT26 cell lines over a period of 6 divisions, corresponding to a 21 day period. (**B**) Fold change of cell proliferation of CT26 and B16-F10 HMGB1^{mock} and HMGB1^{low} cells measured by BrdU incorporation assay. (**C**) Real-time cell proliferation curves of CT26 and B16-F10 HMGB1^{mock} and HMGB1^{low} cells. The timepoints at which the two-way Anova p-value was significant (< 0.05) are highlighted in grey. (**D**) Growth rate calculated from the cell proliferation curves. (**A-D**) Each dot represents one biological replicate, and data is represented as mean ± SD of independent experiments. Unpaired t-test (A-C) and two-way Anova were used, ns: not significant. (**E**) Representative images of HMGB1^{mock} (top) and HMGB1^{low} (bottom) in CT26 (left) and B16-F10 (right) cells, obtained from the Incucyte's imaging system after 48 hours of culture. Scale bars 400 µm

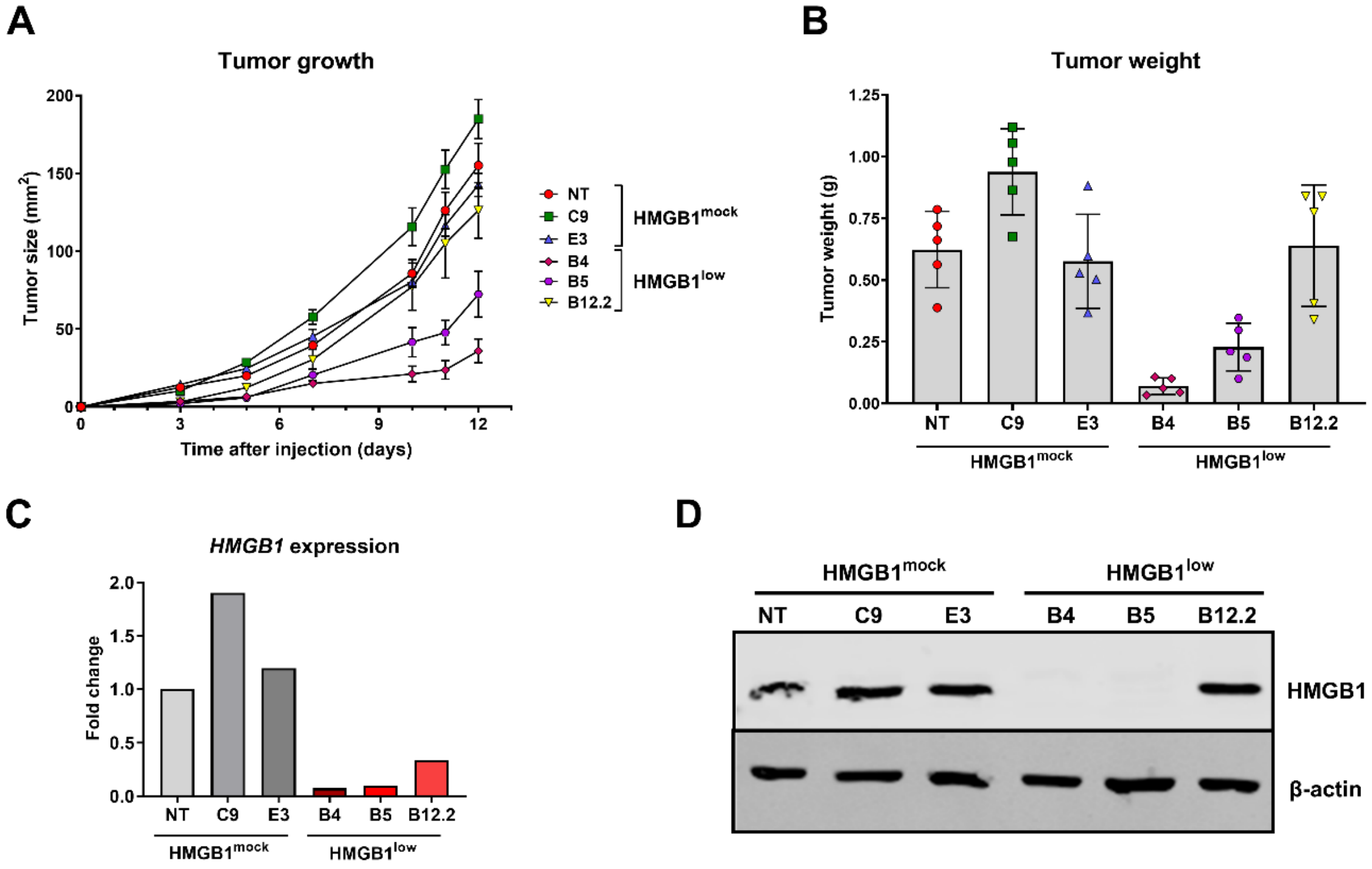

Fig. S3 Downregulation of HMGB1 in CT26 cells reduces tumor growth in several different clones. (**A**) Growth curve and (**B**) endpoint weights of tumors derived from different clones of CT26 $HMGB1^{mock}$ (NT, C9, and E3) and $HMGB1^{low}$ cells (B4, B5 and B12.2). (**C**) Relative expression of *Hmgb1* measured by qRT-PCR on clones from CT26 $HMGB1^{mock}$ and $HMGB1^{low}$ cell lines prior to tumor inoculation. (**D**) Immunoblot of lysates of whole tumors from implanted $HMGB1^{mock}$ and $HMGB1^{low}$ CT26 cell lines probed with HMGB1 (29 kDa) and β-actin (42 kDa) antibodies

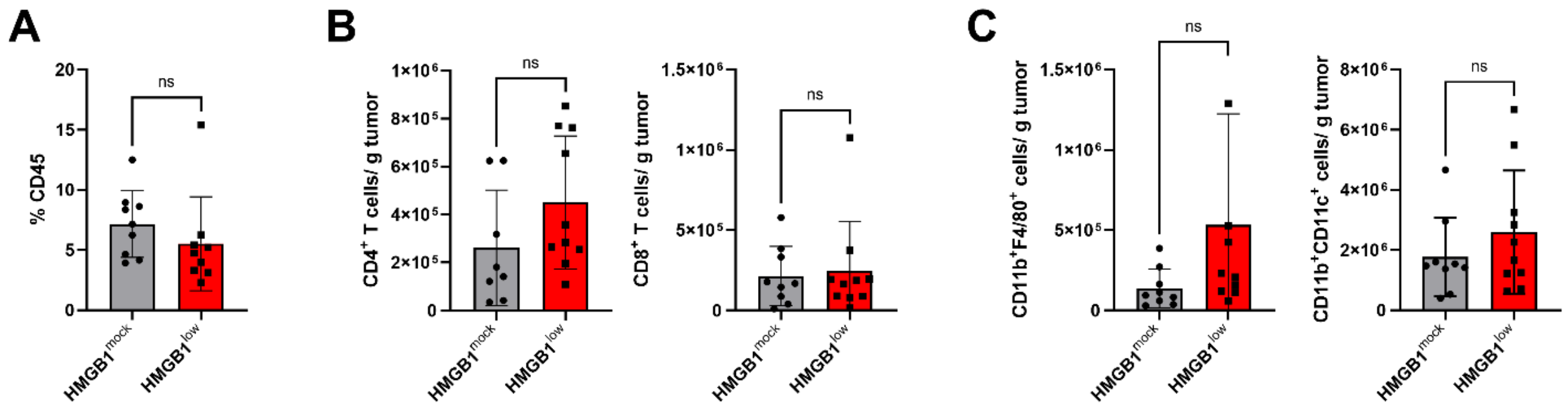

Fig. S4 Immune infiltration profile of subcutaneous B16-F10 HMGB1^{mock} and HMGB1^{low} tumors. (**A**) Percentage of CD45^{+} immune cells, (**B**) absolute numbers of CD4^{+} T cells, CD8^{+} T cells, and (**C**) CD11b^{+}F4/80^{+} macrophages and CD11b^{+}CD11c^{+} dendritic cells (DCs) per gram of tumor tissue analysed by flow cytometry. (**A-C**) Unpaired t-test was used, ns: not significant

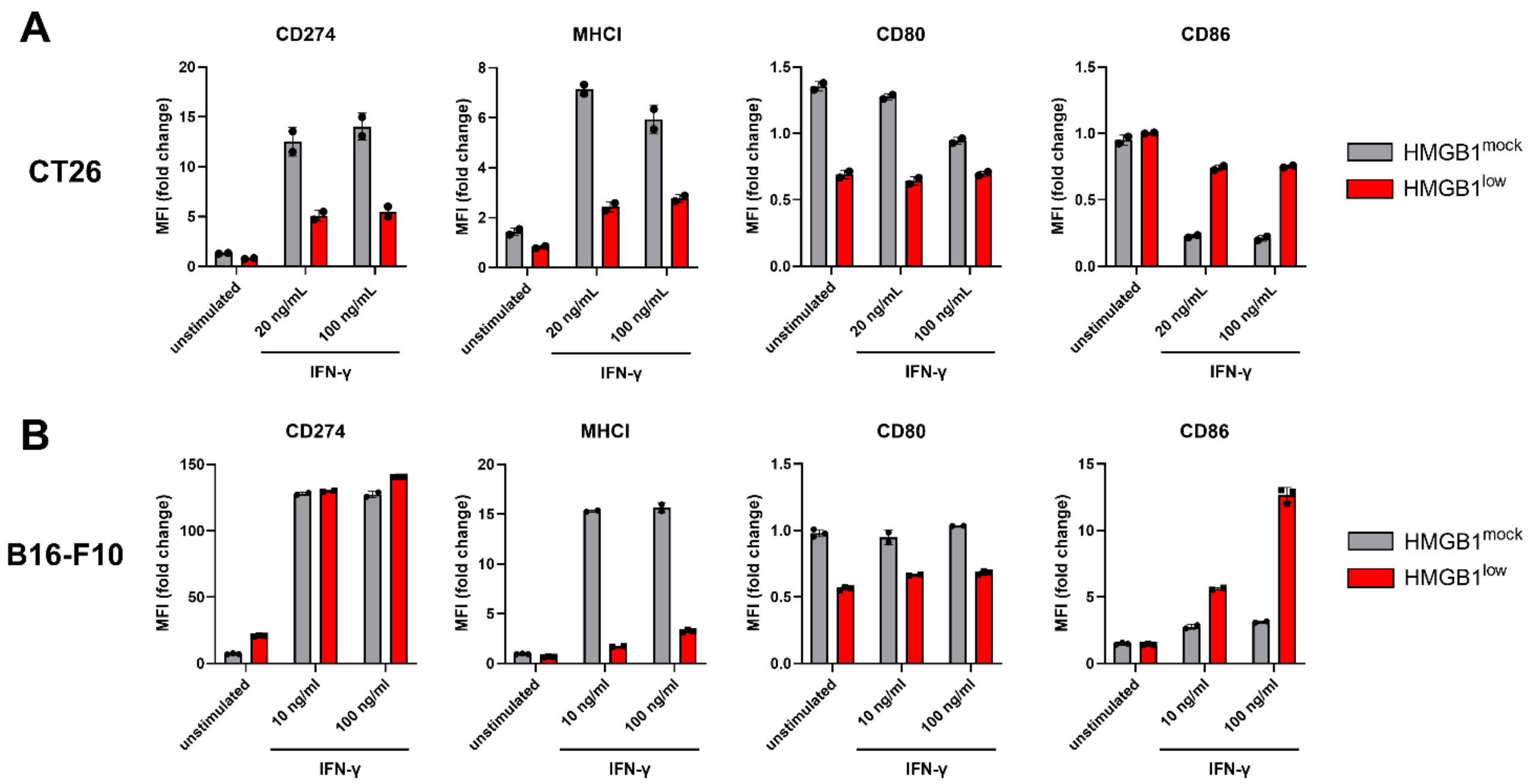

Fig. S5 Immunophenotyping of CT26 and B16-F10 HMGB1^{mock} and HMGB1^{low} cell lines for key molecules of immune interaction. Flow cytometry was used to assess expression of the surface markers CD274, MHC-I, CD80 and CD86 by (**A**) CT26 and (**B**) B16-F10 HMGB1^{mock} and HMGB1^{low} cell lines without or with stimulation by IFN-γ. Mean fluorescent intensity (MFI, fold change with respect to isotype controls) of the indicated markers from corresponding cells are shown. Each dot represents one biological replicate

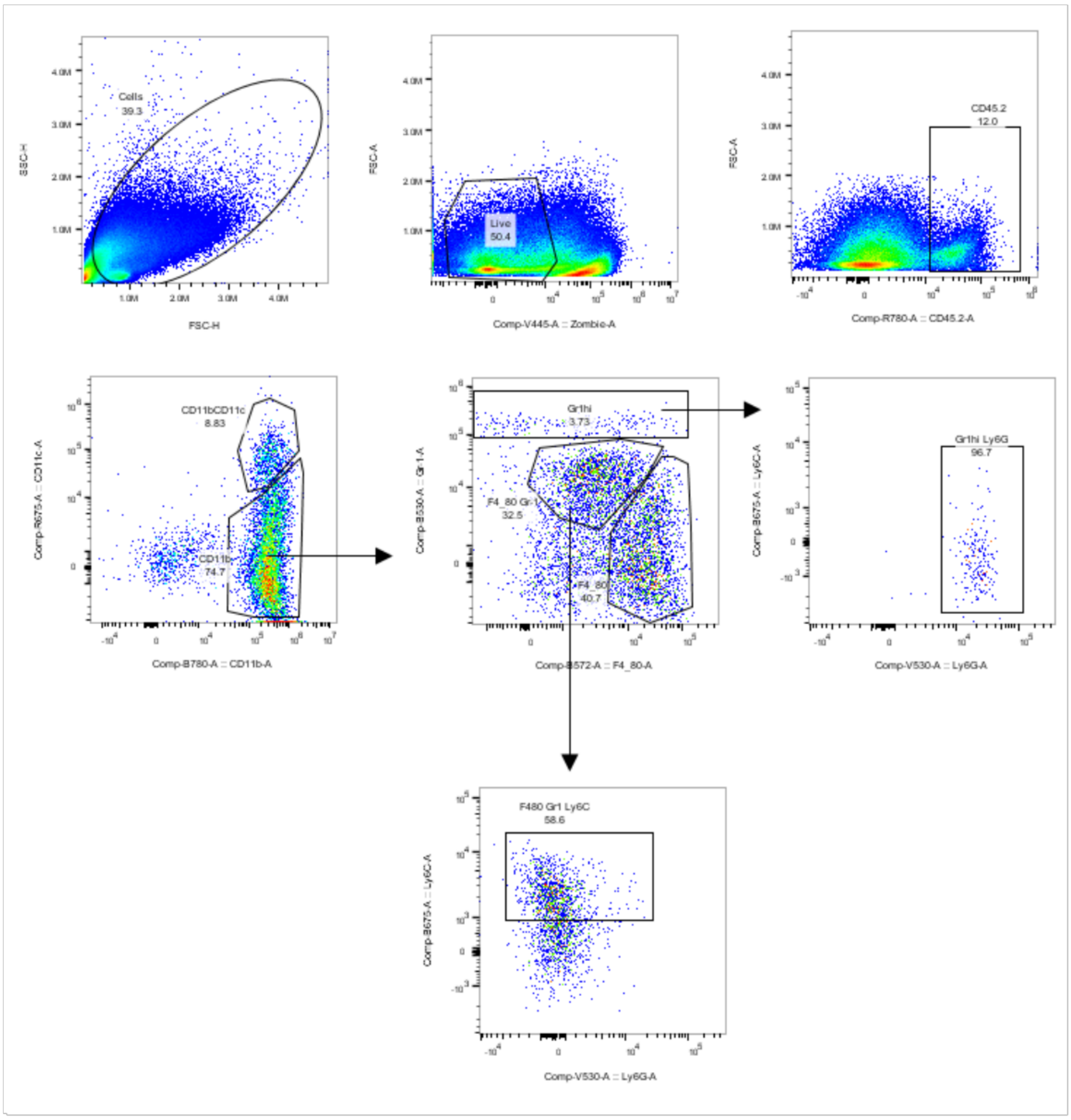

Fig. S6 Gating strategy for analysis of tumors from NSG mice. The first gate was set to exclude cell debris. Dead cells were excluded using viability marker, Zombie-A. The gate on the CD45.2$^+$ population ensured the inclusion of leukocytes only. Different subsets of leukocytes are defined as follows: CD11b$^+$ F4/80$^+$, CD11b$^+$ CD11c$^+$, CD11b$^+$Ly6G$^-$Ly6C$^+$, CD11b$^+$Ly6G$^+$Ly6C$^-$

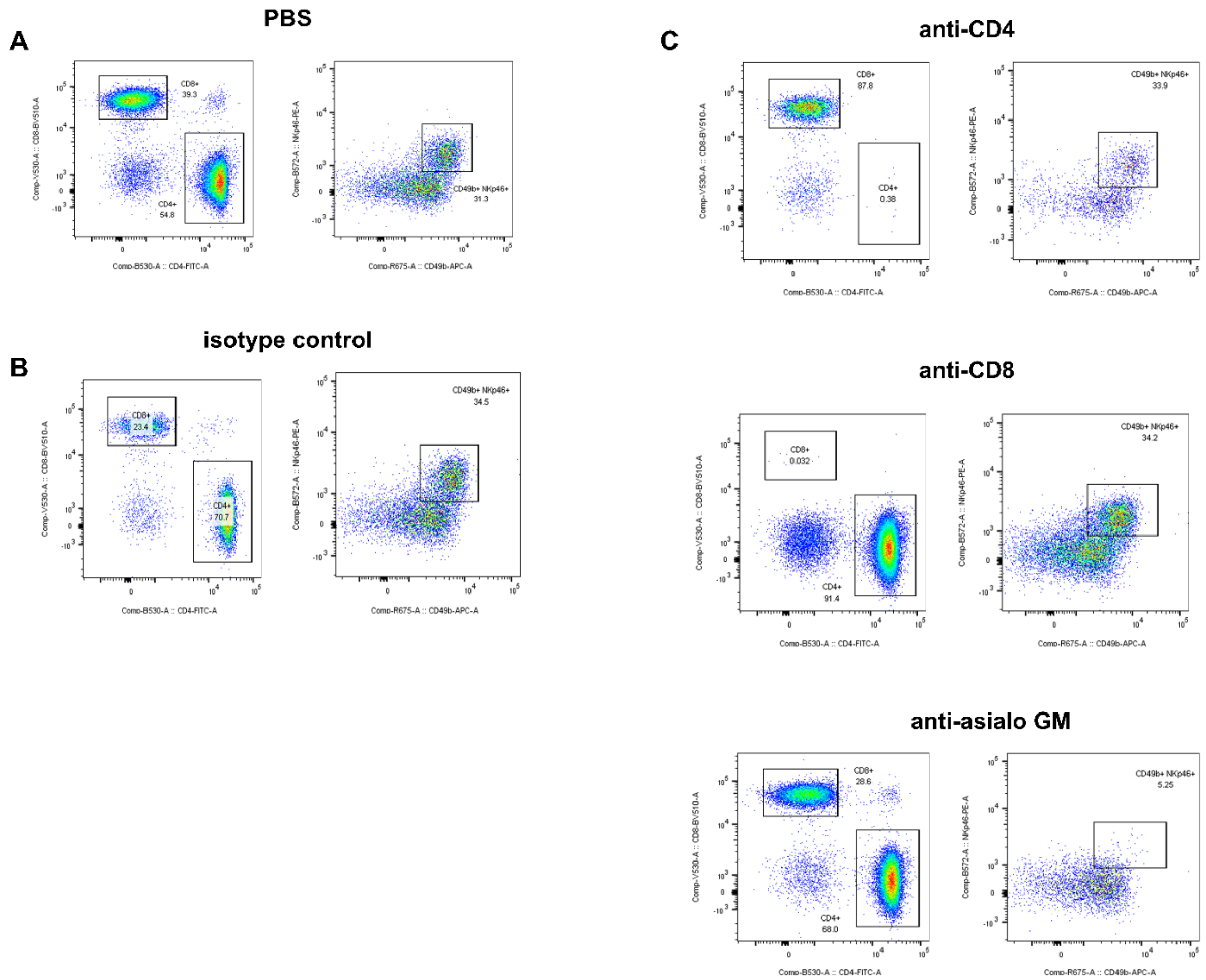

Fig. S7 Efficacy of *in vivo* depletion of CD4$^+$ T cells, CD8$^+$ T cells and NK cells by i.p. injection of monoclonal antibodies. Two days after i.p. injection of anti-CD4, anti-CD8 or anti-asialo GM antibodies, blood was sampled from the tail vein of mice and stained with antibodies against CD4 to determine CD4$^+$ T cell depletion, CD8 to determine CD8$^+$ T cell depletion, and CD49b and NKp46 to determine NK cell depletion. Representative plots for (**A**) PBS, (**B**) isotype control, (**C**) CD4 T-cell, CD8 T-cell and NK cell depletion, respectively

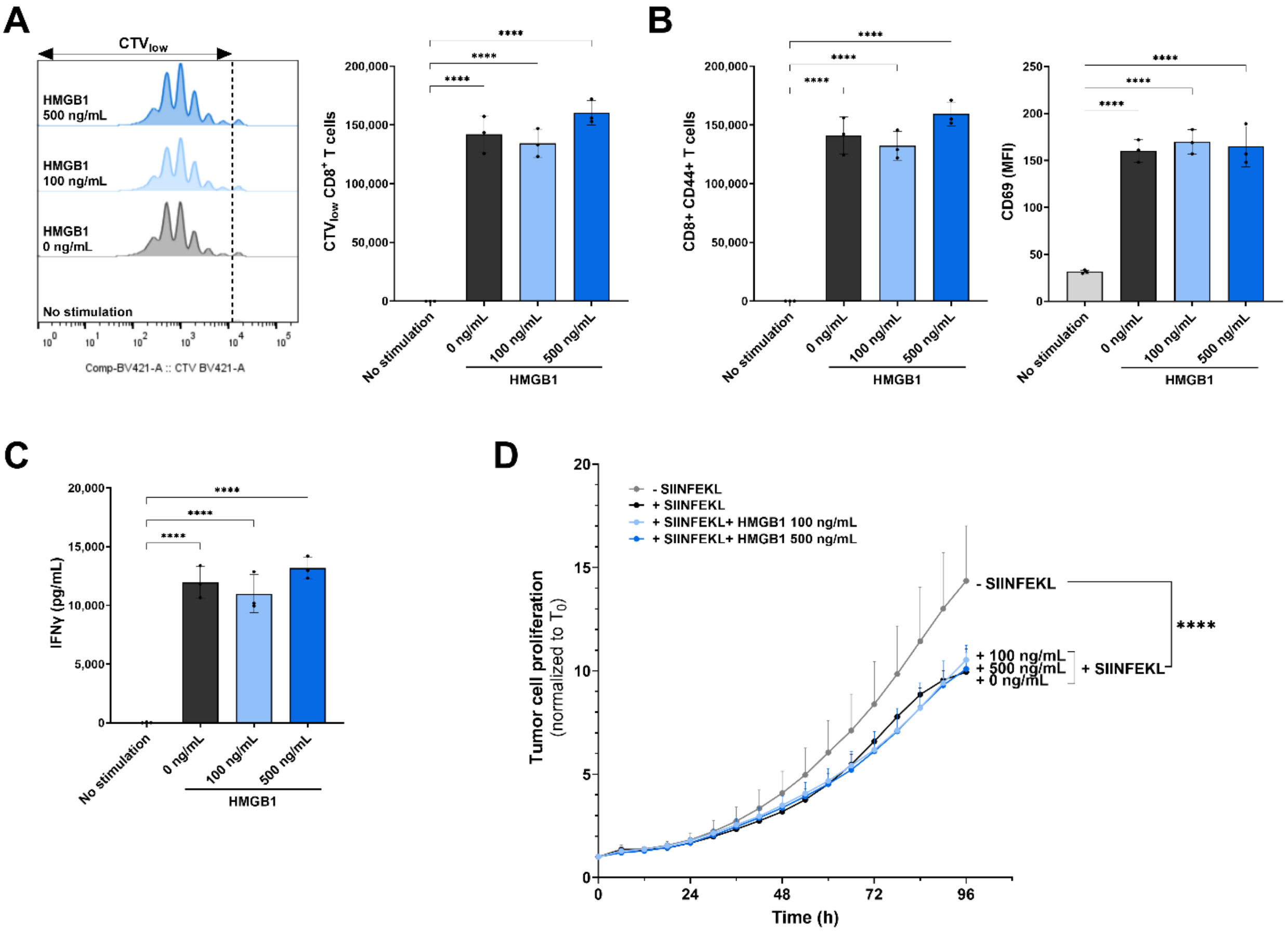

Fig. S8 Effect of extracellular HMGB1 on CD8⁺ T cells function. (**A-C**) $CD8^+$ T cells were treated with recombinant HMGB1 and stimulated with antiCD3/CD28 Dynabeads and IL-2 for 72 hours. (**A**) Proliferation of $CD8^+$ T cells was assessed by flow cytometry based on CellTrace Violet (CTV) dilution and quantification of CTV_{low} $CD8^+$ T cells. (**B**) Number of $CD44^+$ $CD8^+$ T cells and expression of the CD69 surface marker on $CD8^+$ T cells were assessed by flow cytometry (MFI: mean fluorescence intensity). (**C**) IFNγ production was measured by ELISA. (**D**) $CD8^+$ T cells were isolated from OT-I mice and co-cultured with SIINFEKL-labelled cancer cells in the presence or absence of recombinant HMGB1 protein for 96 hours. Co-culture with unlabelled cancer cells (- SIINFEKL) served as a negative control for antigen-specific $CD8^+$ T cell activation. Proliferation of cancer cells was monitored by live-cell imaging to evaluate the cytotoxic activity of $CD8^+$ T cells. (**A-D**) Data are representative of two independent experiments and are depicted as mean ± SD. One-way ANOVA p values (**A-C**) and two-way ANOVA p values (**D**) are shown: *p<0.05, **p<0.01, ****p<0.0001

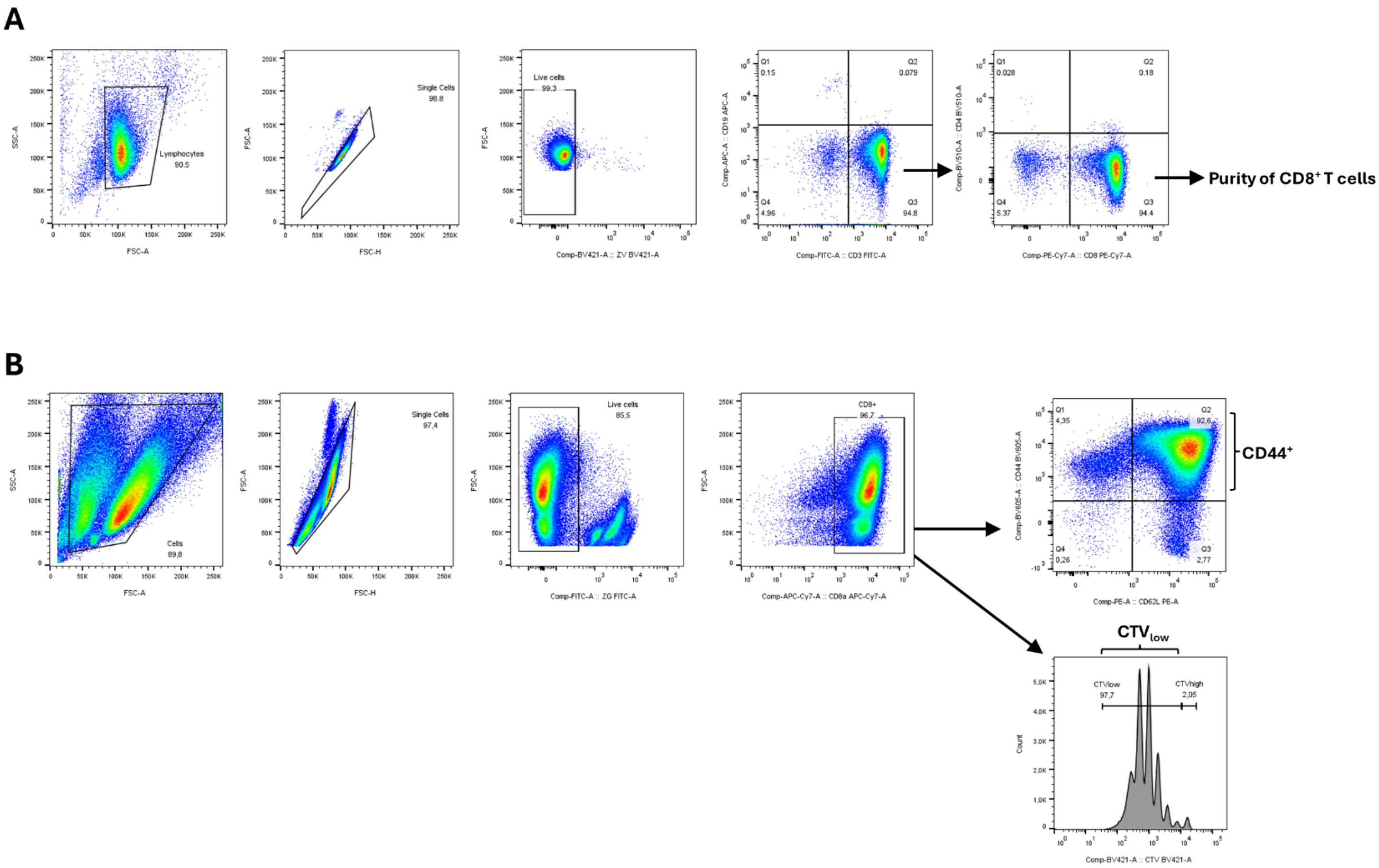

Fig. S9 Gating strategy for analysis of CD8⁺ T cells. (**A**) Gating strategy used to assess the purity of CD8⁺ T cells isolated from splenocytes. (**B**) Gating strategy used to evaluate CD8⁺ T cells activation, including quantification of the CD8⁺ CD44⁺ population and CellTrace Violet (CTV) dilution

Table 1. List of primer pairs.

Gene ID	Forward Primer	Reverse Primer
GAPDH	CAAAGTGGAGATTGTTGCCA	GCCTTGACTGTGCCGTTGAA
HMGB1	TTAGTCCCAGCGAAGGCTAT	CAAGTTTCCTGAGCAATCCA
CXCL12	GCCAACGTCAAGCATCTGAA	CAGCCGTGCAACAATCTGAA
CXCL13	TCTCTCCAGGCCACGGTATTCT	ACCATTTGGCACGAGGATTCAC
CCL2	CCAGCAAGATGATCCCAATG	ACAGACCTCTCTCTTGAGCT
IL6	GTCCTTCCTACCCCAATTTC	GCCGAGTAGATCTCAAAGTG
TNFa	AAATGGCCTCCCTCTCAT	CCTCCACTTGGTGGTTTG
IFNg	AGGAACTGGCAAAAGGATGG	ATGTTGTTGCTGATGGCCTG

Table 2. List of antibodies for Western blot, confocal microscopy and flow cytometry.

Antibody	Source	Identifier/Clone	Antibody	Source	Identifier/Clone
B-actin	Cell Signaling	8H10D10	CD80	Biolegend	16-10A1
CD3	Biolegend	17A2	CD86	Biolegend	GL-1
CD4	Biolegend	GK1.5	CD90.2	Biolegend	30-H12
CD4	Biolegend	RM4-5	CD274	Biolegend	10F.9G2
CD8	Biolegend	53-6.7	CD279	Biolegend	29F.1A12
CD11b	Biolegend	M1/70	F4/80	Biolegend	BM8
CD11c	Biolegend	N418	Foxp3	Biolegend	150D
CD19	Biolegend	6D5	gp70 Tetramer	MBL Int	SPSYVYHQF-PE
CD25	Biolegend	PC61	Gr1	eBioscience	RB6-8C5
CD29	Biolegend	HMB-1	HMGB1	Abcam	ab18256
CD31	Biolegend	MEC13.3	HMGB1	Abcam	ab79823
CD44	Biolegend	IM7	Ly6C	Biolegend	HK1.4
CD45.2	Biolegend	104	Ly6G	Biolegend	1A8
CD49b	Biolegend	DX5	MHC-I	Biolegend	SF1-1.1.1
CD54	Biolegend	YN1/1.7.4	MHC-II	Biolegend	M5/114.15.2
CD62L	Biolegend	MEL-14	NKp64	Biolegend	29A1.4
CD69	Biolegend	H1.2F3			
